# Cryo-EM sample preparation with soft-landing and laser flash melting

**DOI:** 10.1038/s41467-026-77723-6

**Published:** 2026-09-15

**Authors:** Sarah V. Barrass, Tim K. Esser, Nathan J. Mowry, Lukas Eriksson, Jakub Wenz, Laurence T. Seeley, Wyatt A. Curtis, Lindsay A. Baker, Marcel Drabbels, Stephan Rauschenbach, Ulrich J. Lorenz

**Affiliations:** 1 Ecole Polytechnique Fédérale de Lausanne (EPFL), Laboratory of Molecular Nanodynamics, Lausanne, Switzerland; 2 Kavli Institute for Nanoscience Discovery, Dorothy Crowfoot Hodgkin Building, University of Oxford, South Parks Road, Oxford, UK; 3 Department of Chemistry, University of Oxford, Mansfield Road, Oxford, UK; 4 Department of Biochemistry, University of Oxford, South Parks Road, Oxford, UK; 5 Present Address: Thermo Fisher Scientific, De Schakel 2, Eindhoven, Noord-Brabant The Netherlands; 6 Present Address: Ecole Polytechnique Fédérale de Lausanne (EPFL), Laboratory for Ultrafast X-ray Sciences, Lausanne, Switzerland

**Keywords:** Cryoelectron microscopy, Mass spectrometry

## Abstract

The preparation of cryo-EM samples by soft-landing mass spectrometry promises to significantly simplify sample optimization, which has remained an important bottleneck in single-particle cryo-electron microscopy (cryo‑EM). However, only compacted configurations are observed after soft-landing since the proteins are dehydrated in the process. Here, we deposit a layer of amorphous ice onto the sample and briefly flash melt it to rehydrate the soft-landed proteins. Melting with a 30 µs laser pulse creates a broad ensemble of partially rehydrated structures. However, a subset of particles returns to a configuration close to the native state. This allows us to propose strategies to fully rehydrate the entire ensemble and brings routine sample preparation by soft-landing mass spectrometry within reach.

## Abstract (web summary)

Laser flash melting of a cryo-EM sample that is prepared by soft-landing mass spectrometry and subsequent deposition of amorphous ice restores some particles to near-native shape, pointing towards more reliable cryo-EM sample preparation.

## Introduction

Improvements in instrumentation and data analysis are continuing to expand the scope of cryo-EM^1–12^, which is rapidly becoming the preferred method for determining the structure of biological macromolecules. However, obtaining cryo-EM samples suitable for high-resolution imaging has remained a significant bottleneck^13–19^. Contamination of the sample by small molecules frequently reduces the contrast, while the presence of damaged particles or other proteins can make it challenging to obtain high-resolution reconstructions. Samples prepared by plunge freezing may also suffer from an uneven protein distribution across the specimen support as well as variations in ice thickness, with too few areas thin enough for high-resolution imaging^17^. Preparing cryo-EM samples by native soft-landing electrospray ion beam deposition (ESIBD) potentially presents an appealing alternative that should be able to overcome these issues^20–24^. Here, proteins are transferred into the gas-phase via electrospray ionization, mass selected, and gently deposited onto a specimen support held at cryogenic temperature. This offers the advantage that a mass filter can be used to select the protein of interest while removing contaminants and broken particles. Moreover, soft-landing generates a uniform protein distribution across the specimen support. Deposition of a layer of amorphous ice after soft-landing protects the proteins during imaging and improves the attainable spatial resolution. This has recently made it possible to obtain a reconstruction of soft-landed β‑galactosidase at 2.6 Å^25^. However, the proteins are almost entirely dehydrated as they are transferred into the gas phase, so that the soft-landed particles do not present the native protein structure. While secondary structural elements are preserved, water-filled spaces within the proteins usually contract, so that the structures are compacted^26^.

Here, we deposit amorphous ice onto the sample and briefly flash-melt it with a laser beam to rehydrate the soft-landed proteins. We have previously shown that flash melting and revitrifying cryo-EM samples preserves the structure of the proteins, allowing for near-atomic resolution reconstructions from revitrified samples. Moreover, the process can be employed to perform microsecond time-resolved observations of protein dynamics^27–34^. Here, we extend these experiments to rehydrate soft-landed proteins. In a recent publication, this method has been applied to β‑galactosidase particles landed from a molecular ion beam^35^. The experiments showed a change in appearance of the particles, but yielded only a low-resolution reconstruction, making it difficult to assess how the resulting protein structure is related to the native state. Here, we provide a detailed analysis of the rehydration process, showing that flash melting with a 30 µs laser pulse produces a broad ensemble of partially rehydrated structures. Crucially, a subset of the population recovers a configuration close to the native structure, which paves the way for using soft-landing landing electrospray ion beam deposition (ESIBD) as a tool for preparing cryo-EM samples.

## Results

**Fig. 1:**
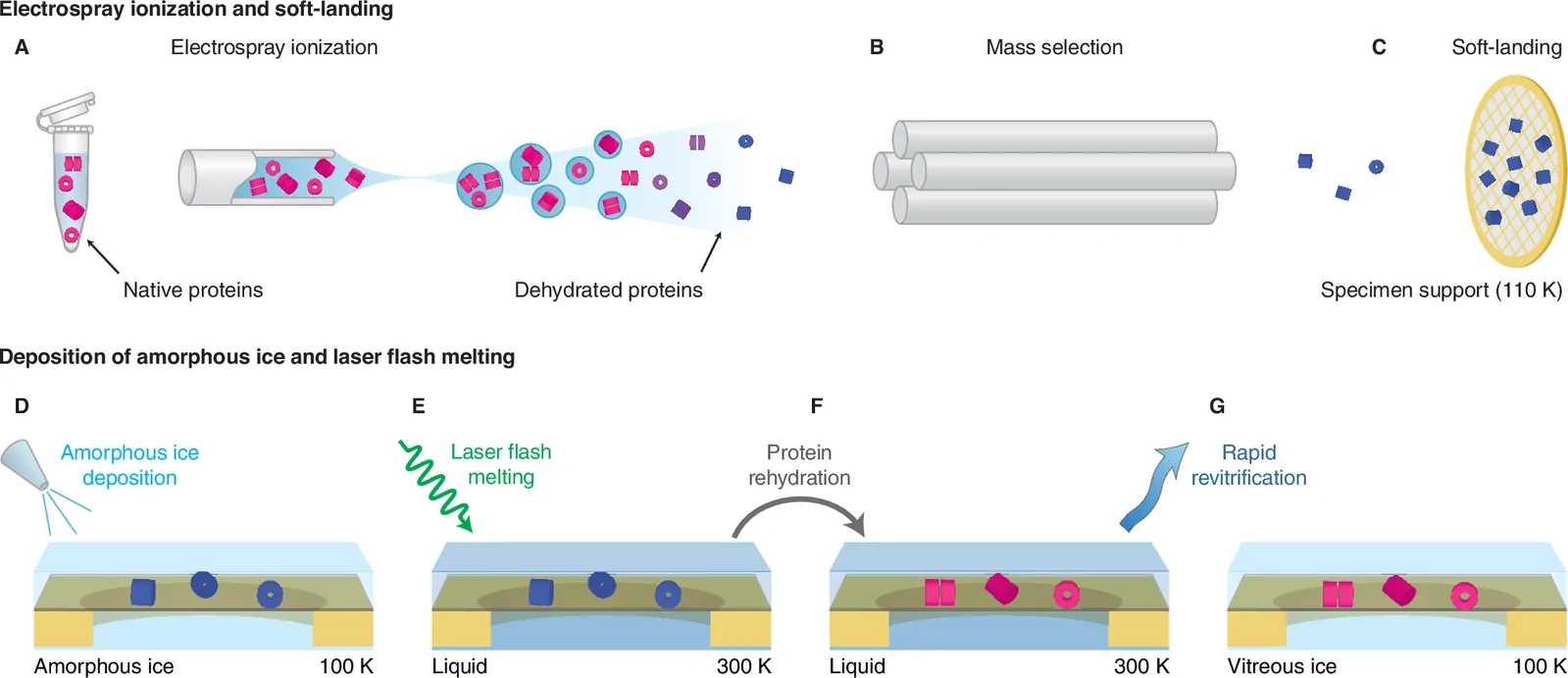
Experimental concept. **A**–**C** Native soft-landing electrospray ion beam deposition (ESIBD). Protein ions are generated by electrospray ionization (**A**), mass selected (**B**), and soft-landed onto an amorphous carbon specimen support held at cryogenic temperature (**C**). In the process, the ions are dehydrated. **D**–**G** Rehydration through deposition of amorphous ice and laser flash melting. Amorphous ice is deposited (**D**), and the sample is laser flash melted (**E**) to allow the proteins to rehydrate (**F**). Once the heating laser is switched off, the sample rapidly cools and revitrifies (**G**).

Figure 1 illustrates the experimental concept. Samples of soft-landed proteins are prepared with a modified mass spectrometer that is equipped with a cryogenic deposition stage (Methods)^25,36^. Proteins (pink) are transferred from solution into the gas phase via native electrospray (Fig. 1A) and enter the vacuum system of the mass spectrometer. During this process, the proteins are dehydrated (blue). The protein ions are mass selected (Fig. 1B) and thermalized in a collision cell to narrow their energy distribution. Finally, they are gently deposited at a collision energy of less than 2 eV per charge onto a 2 nm thick amorphous carbon film on a holey gold specimen support (1.2 µm holes, 1.3 µm apart on a 300-mesh gold grid) held at 110 K (Fig. 1C). To rehydrate the soft-landed proteins, the sample is transferred to a transmission electron microscope modified for time-resolved experiments (Methods)^37,38^. A 50 nm thick layer of amorphous ice is deposited in situ by leaking water vapor into the volume surrounding the sample, which is held at a temperature of 100 K (Fig. 1D). The sample is then flash melted with a 30 µs laser pulse (Fig. 1E, 532 nm wavelength), allowing the proteins to rehydrate (Fig. 1F). Once the heating laser is switched off, the sample rapidly cools and revitrifies within microseconds^27^, trapping the proteins in their new configurations (Fig. 1G). Finally, the sample is transferred to a high-resolution cryo-electron microscope for imaging.

**Fig. 2:**
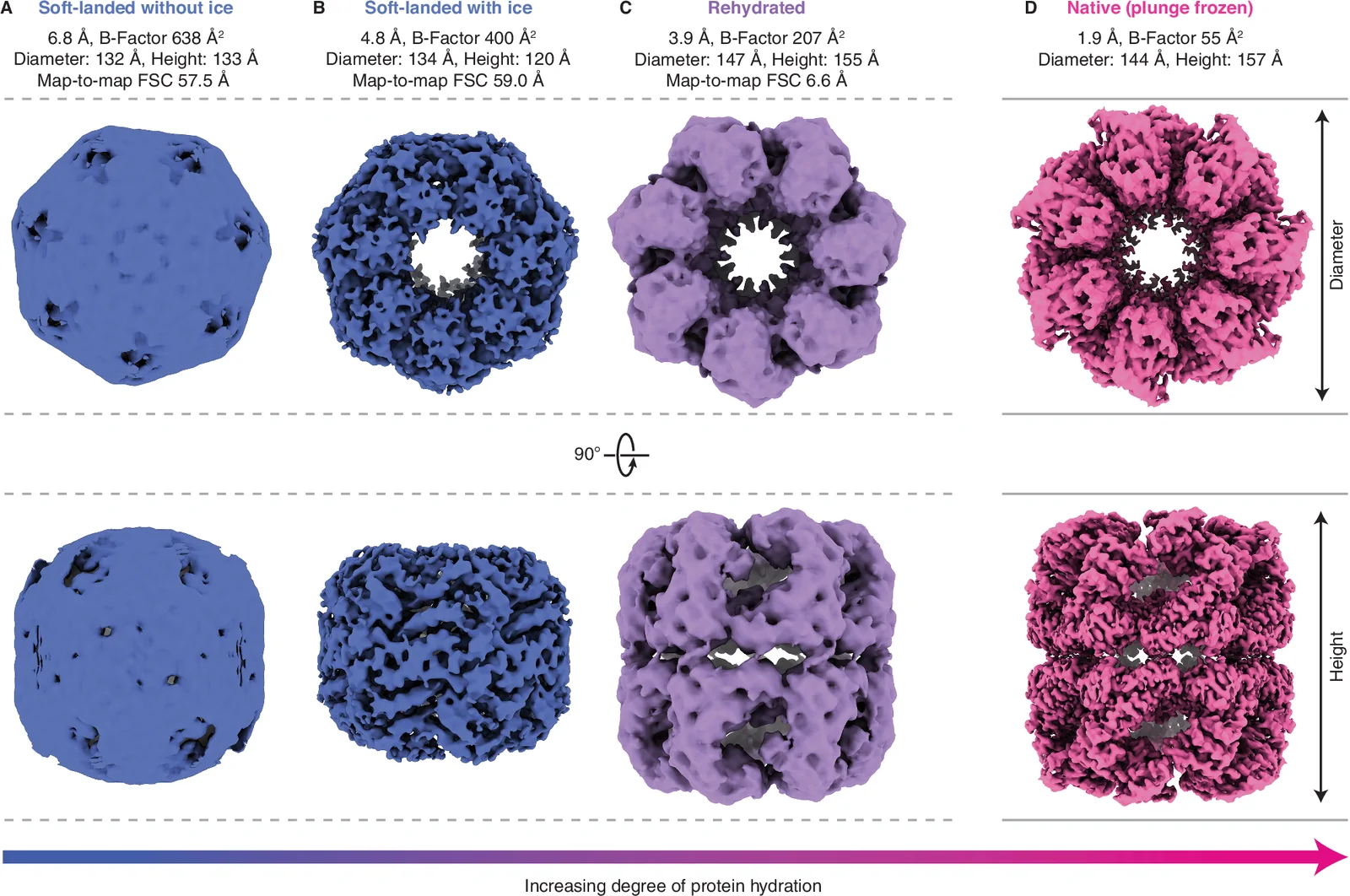
Single-particle reconstructions of soft-landed, rehydrated, and native GroEL. **A**–**D** Maps of GroEL (D_7_ symmetry) after soft-landing (**A**), embedded in amorphous ice (**B**), and after laser melting and rehydration (**C**) are compared with a map of native GroEL from a plunge-frozen sample (**D**). Note that the nominal resolution of the reconstruction in (**A**) does not seem to accurately describe the true resolution. The resolution, B-factor, and dimensions are indicated, as well as the map-to-map FSC with the reconstruction from the plunge frozen sample (Supplementary Information C, threshold 0.5). Note that for the compacted structures in (**A**) and (**B**), the map-to-map FSC is not meaningful.

Figure 2 compares reconstructions of soft-landed and rehydrated GroEL with a reconstruction from a conventional, plunge-frozen sample (Methods, Supplementary Information A, B). While the soft-landed particles only yield a low-resolution reconstruction (Fig. 2A, nominal resolution of 6.8 Å), depositing an additional layer of amorphous ice ( < 10 nm thickness) protects the proteins during imaging and improves the resolution (Figs. 2B, 4.8 Å). A comparison with the native structure (Figs. 2D, 1.9 Å) reveals that dehydration has significantly compacted the soft-landed particles, reducing their height and diameter by about 24% and 7%, respectively. The water-filled spaces between the subunits that are present in the native state have contracted, with only the central cavity of the protein still visible. In contrast, depositing a 50 nm layer of amorphous ice and flash melting and revitrifying the sample with a 30 µs laser pulse allows the soft-landed particles to rehydrate and expand (Fig. 2C). The water-filled spaces between the subunits are restored, and a map yields dimensions close to that of the native structure. The map resolution of 3.9 Å is lower than in a conventional, plunge-frozen sample. Moreover, the Fourier Shell Correlation (FSC) with the map of the native sample yields only a value of 6.6 Å at a threshold of 0.5 (Supplementary Information C). This suggests that the rehydrated ensemble has significant conformational heterogeneity and that during the time window provided by the 30 µs melting laser pulse, most particles have not fully rehydrated. This is also supported by a joint variability analysis of the rehydrated and native particles, which shows that their distributions are separated, with only some overlap at the wings (Supplementary Fig. 9).

**Fig. 3:**
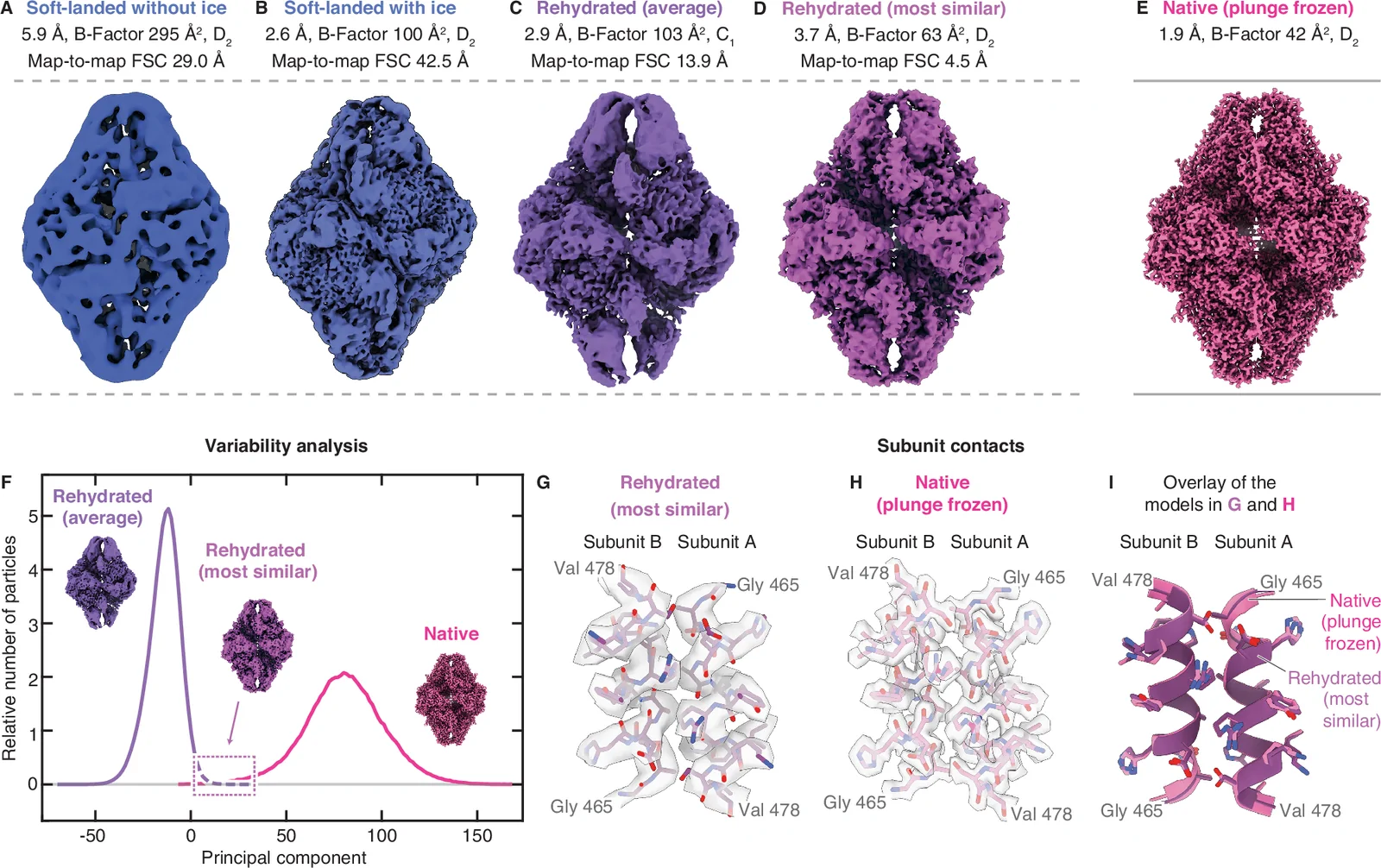
Single-particle reconstructions of soft-landed, rehydrated, and native β-galactosidase. **A**–**E** Maps of β-galactosidase after soft-landing (**A**), embedded in amorphous ice (**B**, EMD-18244^25^), and the average structure obtained after laser melting and rehydration (**C**) are compared with a map of the native structure from a plunge-frozen sample (**E**). The volume in (**D**) represents a reconstruction of the rehydrated particles that most closely resemble the native state, as determined from the variability in (**F**). All reconstructions are performed with D_2_ symmetry, with the exception of the map in (**C**), for which no symmetry was applied. The resolution, B-factor, and symmetry are indicated, as well as the map-to-map FSC with the reconstruction from the plunge frozen sample (Supplementary Information C, threshold 0.5). Note that for the compacted structures in (**A**) and (**B**), the map-to-map FSC is not meaningful. **F** Joint variability analysis of the rehydrated (purple) and native particles (pink). The particle distributions are shown as a function of the first principal component. A box marks the subset of rehydrated particles that were used for the reconstruction in (**D**). **G**–**I** Comparison of the subunit contacts in the maps of the most similar rehydrated particles (**G**) and native particles (**H**), displayed at 4 σ and 8 σ above the mean, respectively. The corresponding molecular models closely resemble each other as revealed by an overlay in (**I**).

A soft-landing and rehydration experiment with β-galactosidase^25^ provides a more detailed understanding of the rehydration process and reveals that laser melting is able to return a subset of the particles close to their native state (Fig. 3, Methods, Supplementary Information A, B). Similarly to GroEL, the bare, soft-landed β-galactosidase particles only yield a low-resolution reconstruction (Fig. 3A). While depositing amorphous ice improves the resolution (Fig. 3B), it is not able to return the particles to their native structure (Fig. 3E). In contrast, flash melting with a 30 µs laser pulse allows the proteins to rehydrate and expand (Fig. 3C). However, the resulting map appears slightly twisted compared to the native state, with parts of the protein surface exhibiting a low resolution (Supplementary Fig. 7D). Variability analysis^39^ of the combined rehydrated and native particles confirms that laser flash melting has not allowed most particles to fully return to their native structure (Supplementary Information D). Figure 3F displays the particle distributions along the first variability component, which is largely associated with the expansion of the particles as they rehydrate. The ensemble of the rehydrated particles (purple) is separated from the native particles (pink), with only the tail of the distribution overlapping with the native ensemble. If we select the particles from this tail, which are most similar to the native state (about 25’000 particles, marked with a dashed box), we obtain the reconstruction in Fig. 3D (3.7 Å). As illustrated in Supplementary Fig. 12, this reconstruction resembles the native structure, demonstrating that during the short time window provided by the melting laser pulse, a subset of the particles returns to a configuration close to the native structure. This is also reflected in the similarity of the molecular models that we have built for both structures (Supplementary Information E and Supplementary Fig. 14, root-mean-square deviation of 0.7 Å), as illustrated in Fig. 3G–I for the subunit contacts within the core of the protein. Note that we can obtain a reconstruction very similar to that in Fig. 3D without including native particles in the variability analysis as a reference (Supplementary Information D).

While the reconstruction of the rehydrated particles most similar to the native state (Fig. 3D) resembles the map obtained from a plunge-frozen sample (Fig. 3E), it features a large variation in the local resolution (Supplementary Fig. 8E). Whereas the core of the protein exhibits the highest resolution, parts of the periphery and particularly the protein surface are less well-resolved, indicating larger conformational heterogeneity than in a conventional cryo‑EM sample, where the particles are fully equilibrated. This is consistent with the B‑factor of 63 Å^2^ being larger than for a reconstruction from a conventional sample (B‑factor of 42 Å^2^). For comparison, the reconstruction from all rehydrated particles has a B-factor of 103 Å^2^ (a reconstruction with C_1_ symmetry of the particles most similar to the native state has a B-factor of 41 Å^2^, Supplementary Fig. 15). The map-to-map FSC of the reconstruction in Fig. 3D with the reconstruction of the native state yields a value of 5.7 Å at a threshold of 0.5, compared with 16.9 Å for the average structure containing all particles (Supplementary Information C; for comparison, map-to-model FSCs are listed in Supplementary Table 1). This indicates that some distortions of the protein structure remain even for the rehydrated particles that the variability analysis identifies as most similar to the native state. A local FSC of the map of the most similar rehydrated particles with the map from a plunge-frozen sample reveals that some of the largest structural differences occur near water-filled voids, suggesting that they have not yet fully re-expanded (Supplementary Fig. 13).

**Fig. 4:**
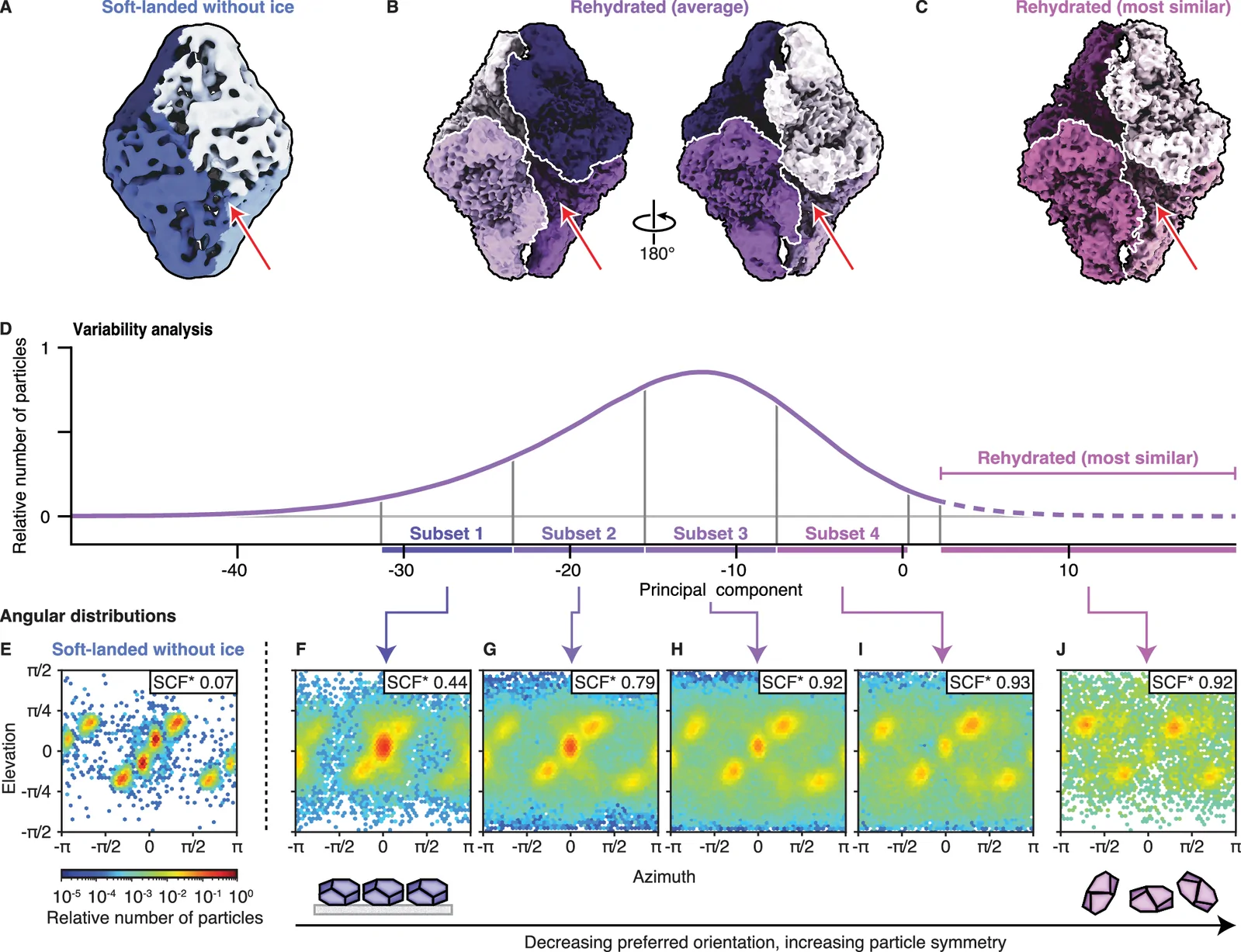
Adsorption to interfaces slows rehydration and causes the particles to adopt asymmetric configurations. **A**–**C** Reconstructions of β-galactosidase for the bare, soft-landed particles (**A**, from Fig. 3A), all rehydrated particles (**B**, from Fig. 3C), and the rehydrated particles most similar to the native structure (**C**, from Fig. 3D). The subunits are highlighted in colors. The average structure of the rehydrated particles in (**B**) shows significant asymmetry in the expansion of its two faces (arrows). **D** The central part of the distribution of rehydrated particles along the variability component in Fig. 2F is divided into four subsets. The particles included in the reconstruction in (**C**) are also indicated. **E**–**J** Angular distributions of the bare, soft-landed particles (**E**), of the four subsets of rehydrated particles (**F**–**I**), and of the rehydrated particles most similar to the native state (**J**). The angular distributions of the rehydrated particles are shown for one of two samples, with the other presented in Supplementary Fig. 16. The sampling compensation factor (SCF*), a measure of the degree of preferred orientation, is indicated. Along the principal component, preferred particle orientation decreases, and the particles become more symmetric (Supplementary Fig. 16), suggesting that adsorption causes the particles to rehydrate asymmetrically.

A detailed analysis of the conformational ensemble of the rehydrated particles suggests that adsorption at an interface slows the rehydration process, causing the particles to adopt asymmetric configurations. When the bare, soft-landed particles (Fig. 4A) are rehydrated, their faces expand, with a gap between the subunits widening, as seen in the average structure of the rehydrated particles (Fig. 4B, highlighted with arrows). Close inspection reveals that this expansion is asymmetric, with one face of the structure more expanded than the other. We find that this asymmetry decreases along the first principal component of the variability analysis of Fig. 3F, with the particles becoming more symmetric the closer they are to the native state (Supplementary Fig. 16, the motion is also illustrated in Supplementary Movie 1). The rehydrated particles most similar to the native structure barely show any asymmetry (Fig. 4C, see Supplementary Fig. 15 for a reconstruction with C_1_ symmetry). Remarkably, the decrease in asymmetry along the principal component is accompanied by a decrease in the preferred orientation of the particles. This can be seen if we divide the central portion of the distribution of the rehydrated particles into four subsets as illustrated in Fig. 4D and determine the angular distribution for each (Fig. 4F–I, shown for one of two samples from which data were recorded, as detailed in Supplementary Fig. 16). The particles in subset 1, which are the most different from the native structure and the least symmetric, exhibit the most pronounced preferred orientation (Fig. 4F), with one particle view predominantly populated that corresponds to the viewing direction in Fig. 4B (left).

Preferred orientation results from the adsorption of particles to an interface of the cryo-EM sample^40–42^. Evidently, most particles in subset 1 are adsorbed with one of their faces, which strongly suggests that this slows the expansion of this face, breaking the symmetry of the particles as they rehydrate. Along the principal component, particles adsorbed in this orientation become less frequent (Fig. 4G–I). Moreover, the angular distribution turns more homogeneous, with the sampling compensation factor (SCF*), a measure of the degree of preferred orientation, improving from 0.44 in subset 1 to 0.93 in subset 4. The SCF* takes values between zero and one, with one corresponding to a perfectly isotropic angular distribution. The rehydrated particles that are most similar to the native state and most symmetric show little preferred orientation, with an SCF* of 0.92 (Fig. 4J). Their angular distribution shows maxima that are only sparsely populated in subset 1, suggesting that adsorption in these orientations allows the particles to rehydrate more easily.

Our results suggest that adsorption to an interface hinders many particles from fully returning to the native state during the short time window provided by the melting laser pulses. It is conceivable that some particles simply remain adsorbed to the amorphous carbon film of the specimen support onto which they were soft-landed. It is also possible that once the sample is liquid, other particles quickly diffuse to the air-water interface (estimated diffusion time on the order of 10 µs) and adsorb there. The angular distribution of the most asymmetric particles suggests that both scenarios may occur. It features a second, less pronounced maximum that corresponds to the opposite viewing direction (Fig. 4F and Supplementary Fig. 16C), suggesting that particles also adsorb to the opposite sample interface. It is difficult to determine which interface the particles predominantly adsorb to merely based on the preferred orientation of the most asymmetric particles, since it resembles both that of the bare, soft-landed particles (Fig. 4E) and that of the particles in a conventional cryo-EM sample, where adsorption to the air-water interface is likely dominant (Supplementary Fig. 5F). It should however be possible to distinguish between both scenarios with cryo-electron tomography or by taking care to mount the sample in a well-defined orientation during image acquisition, which was not done here.

In conclusion, our experiments outline a path towards preparing cryo-EM samples with soft-landing mass spectrometry by depositing amorphous ice onto the soft-landed particles and using laser flash melting to rehydrate them. This approach allows us to prepare samples with a uniform particle distribution across the specimen support. While a conventional sample of β‑galactosidase contains some fraction of dimers (Supplementary Fig. 5C), only tetramers are found after soft-landing (Supplementary Figs. 6, 7), underlining the potential of this approach to reduce sample heterogeneity. We observe that during the time window provided by a 30 µs melting laser pulse, most particles only partially rehydrate, which results in a broad conformational distribution. Our experiments reveal details of the rehydration process and thus open up avenues for studying protein hydration. Moreover, they show that for β‑galactosidase, a subset of particles does return to a configuration close to the native state. This suggests that it should ultimately be possible to fully rehydrate all particles simply by providing them with more time to do so.

We have recently demonstrated that the time window during which the laser pulse keeps the sample liquid can be extended from tens to hundreds of microseconds by enclosing the cryo-EM sample between two ultrathin silicon dioxide layers^33^. These sealing membranes, which we vapor deposit onto the sample, stabilize the thin liquid film under laser irradiation and prevent its evaporation. For compact proteins such as β‑galactosidase, this should provide enough time for most particles to fully rehydrate. Our experiments also suggest that the rehydration process can be accelerated if the adsorption of the particles to interfaces can be reduced. As we have recently shown, the hydrophilic silicon dioxide membranes eliminate interactions of the particles with the hydrophobic air-water interface^33^. It should also be possible to minimize interactions with the support film by optimizing the choice of material or by depositing a thin layer of amorphous ice prior to soft-landing. Future experiments will also have to show whether this approach can be generalized to a wide range of proteins. For example, some particles require the presence of specific ions or buffer components to maintain their native fold. For such particles, it should be possible to co-deposit these compounds in the desired stoichiometry. It is also well known that some particles cannot be lyophilized and successfully rehydrated, as they misfold or aggregate in the process, which raises the question whether they would be amenable to our approach. It remains to be seen if the rapid dehydration that the electrosprayed particles experience is fast enough to outrun slower irreversible structural changes and aggregation. Note that dehydration is avoided in approaches in which the electrosprayed particles are deposited on the grid in small droplets^43^. However, such approaches cannot benefit from the ability to mass filter the particles to remove contaminants, as well as the uniform particle distribution that is obtained with soft landing. In the future, it may even become possible to use ion mobility spectrometry to select specific conformational states.

Finally, our experiments also suggest an approach for initiating dynamics in microsecond time-resolved cryo-EM experiments^34^. It should be possible to use soft-landing mass spectrometry to deposit a ligand or small molecule onto a cryo-EM sample, so that upon laser flash melting, rapid mixing occurs, and the compound can interact with the embedded proteins to initiate dynamics. This provides the advantage that one would not be limited to the availability of suitable photocaged compounds in order to initiate dynamics and should open up possibilities for studying signaling, drug binding, or even protein-protein interactions.

## Methods

### Preparation of protein solutions

Samples of GroEL and β-galactosidase were purchased from Sigma-Aldrich (G3153-5MG and C7688-1MG). For the preparation of conventional cryo-EM samples, solutions were prepared as follows. The lyophilized GroEL powder was resuspended in 20 mM Tris (pH 7.9), 50 mM KCl, 0.5 mM EDTA, 5 mM MgCl_2_, and 0.5 mg/mL ATP to give a protein concentration of 4 mg/mL. A solution of β-galactosidase (1.35 mg/mL) was prepared by resuspending in 25 mM Tris (pH 7.9), 50 mM NaCl, 10 mM EDTA, and 2 mM MgCl_2_. For native mass spectrometry, the GroEL solution was desalted by passing it through two P6 buffer exchange columns (7326222, Biorad) that had been equilibrated with 200 mM ammonium acetate (pH 6.9). A solution of β-galactosidase (10 mM) was obtained by dissolving the lyophilized powder in 200 mM ammonium acetate and desalting with the same procedure.

### Native mass spectrometry and ion beam deposition onto specimen supports

Native mass spectrometry was performed on a commercial mass spectrometer (Thermo Scientific Q Exactive UHMR instrument controlled by Thermo Fisher Scientific Exactive Series Tune v2.10) that was modified for ion beam deposition experiments^20,25,36^. The following operating parameters were typically used.

**Table:** 

Source DC offset	21 V
S-lens RF level	200 (300 V_p-p_)
Transfer capillary temperature	60 °C
Ion transmission settings	“high m/z” (700 V_p-p_ for the injection flatapole and 900 V_p-p_ for the bent flatapole, transfer multipole, and collision cell)
Detector optimization	“high m/z”
Injection flatapole	5 V
Interflatapole lens	4 V
Bent flatapole	2 V
Transfer multipole	0 V
Collision-cell pressure setting	7 (N_2_)
Collision-cell multipole DC	− 5 V
Collision-cell exit lens	− 15 V

Nano-electrospray ionization emitters were fabricated by pulling borosilicate glass capillaries (30-0042, Harvard Bioscience) with a pipette puller (P-1000, Sutter Instrument) and gold coating them with a sputter coater (108 A/SE, Cressington). Up to 10 µl of protein solution was loaded into an emitter, and the emitter tip was clipped to create an opening of 0.1 to 10 µm^44^. Electrospray ionization was initiated by applying a potential of 1.2 kV and a gentle nanoflow pressure ( < 200 mbar above atmospheric pressure).

While the conventional operating mode of the mass spectrometer was used to collect mass spectra (Thermo Fisher Scientific Xcalibur Version 4.2.28.14)^45^, a modified scan matrix was employed for ion deposition that allows the ions to pass through the C-trap and collision cell without being trapped and reach the deposition stage. The number of water molecules attached to the protein complex ions under these conditions is estimated to be less than 200, as determined by comparing with mass spectra recorded after desolvating the ions in the collision cell. Note that the protein complex ions may also contain residual salt.

Protein complex ions were soft-landed on specimen supports as previously described^20,25,36^. Clipped UltrAuFoil specimen grids covered with a 2 nm thick layer of amorphous carbon (R1.2/1.3, 300 mesh, Quantifoil) were mounted in a shuttle, which was inserted into the deposition stage of the modified mass spectrometer^25^ a few minutes before the experiment. The deposition stage is cooled to a temperature of 110 K by a closed-cycle helium cryo-cooler (Sunpower Cryotel GTLT). When the shuttle is inserted, a spring-loaded mechanism opens a cover, exposing the grid for the ion beam deposition. At the same time, an electrical contact is created, which is used to control the sample potential and, with it, the deposition energy. This contact is also used to monitor the ion deposition current with a picoammeter, so that the protein coverage on the specimen grid can be controlled. Protein ions were deposited with an energy of 2 eV per charge, as measured with a retarding-grid energy detector, with a typical ion current of 50 pA and 20 min deposition time. The deposition is performed in ultrahigh vacuum ( < 10^−9^ mbar).

For the reconstructions in Figs. 2B, 3B (GroEL and β-galactosidase, “soft-landed with ice”), a < 10 nm thick layer of amorphous ice was additionally deposited onto the specimen grids after ion beam deposition. To this end, the partial pressure of water vapor in the deposition chamber was raised to 5･10^-5 ^mbar using a gas dosing valve, while the sample temperature was maintained between 111 K and 114 K. After a typical deposition time of about 1 min, the partial pressure of water was reduced by more than 2 orders of magnitude, and the sample was cooled to about 95 K to prevent devitrification during sample transfer. Finally, the grids were transferred into liquid nitrogen in under 2 min, as previously described^25^. The deposition rate was calibrated separately by depositing layers of amorphous ice under identical conditions and determining their thicknesses with electron tomography^26^.

### Laser melting and revitrification experiments

Laser melting and revitrification experiments were performed with a modified JEOL 2010F transmission electron microscope^37,38^. Microsecond laser pulses for flash melting (30 µs, 532 nm wavelength, 83–149 mW laser power) were generated by chopping the output of a continuous laser (Coherent Verdi G7) with an acousto-optic modulator (AA Opto-Electronic) controlled by an arbitrary function generator (Tektronix AFG1062). The laser beam was focused to a spot size of 38 μm FWHM on the sample, as determined from an image of the laser beam recorded with a CCD camera placed in a conjugate plane. The laser beam was centered onto a grid square in order to melt and revitrify a sample area.

Samples prepared by soft-landing (no additional ice coverage) were loaded into the microscope with a Simple Origin Dual Autogrid cryo specimen holder, 100 K sample temperature. Amorphous ice was then deposited onto the sample in situ by leaking water vapor into the area surrounding the sample with a gas dosing valve as previously described^37,38^. With the shutter of the cryo specimen holder still covering the sample to prevent any deposition of amorphous ice, the gas dosing valve was opened, and the deposition rate was set to about 15 nm/min (calibrated in a separate experiment as described before^37,38^). After the deposition rate had been stable for 10 min, the shutter was opened to deposit a 50 nm thick layer of amorphous ice (25 nm on each side). The deposition was stopped by covering the sample again with the shutter. The gas dosing valve was then closed, and at least one hour was allowed for the column of the microscope to return to its base pressure before the shutter was opened again to perform the laser revitrification experiments.

### Preparation of conventional cryo-EM samples

Conventional cryo-EM samples of GroEL and β-galactosidase were prepared after centrifuging the protein solutions to remove aggregates (15 min, 18000 × *g*, 4 °C). For GroEL, 3 µL of the protein solution was applied to holey amorphous carbon grids (R2/1, 200 mesh copper, Quantifoil) that had been rendered hydrophilic through glow-discharging. The grids were blotted for 5 s and plunged into a liquid ethane/propane mixture using a manual plunger. Cryo-EM samples of β‑galactosidase were prepared with the same procedure, using a volume of 4 µL, holey carbon grids covered with a 2 nm thick amorphous carbon layer (R2/1, 400 mesh copper, Quantifoil), and liquid ethane/propane as a cryogen.

### Reporting summary

Further information on research design is available in the Nature Portfolio Reporting Summary linked to this article.

## Supplementary information

Supplementary Information

Description of Additional Supplementary Files

Supplementary Movie 1

Reporting Summary

Transparent Peer Review file

## Data Availability

Unless otherwise stated, all data supporting the results of this study can be found in the article, supplementary, and source data files. The cryo-EM maps have been deposited in the Electron Microscopy Data Bank (EMDB) under accession codes EMD-52627 (soft-landed GroEL without ice) [https://www.ebi.ac.uk/emdb/EMD-52627], EMD-52626 (soft-landed GroEL with ice) [https://www.ebi.ac.uk/emdb/EMD-52626], EMD-52284 (soft-landed and rehydrated GroEL) [https://www.ebi.ac.uk/emdb/EMD-52284], EMD-52282 (plunge-frozen GroEL) [https://www.ebi.ac.uk/emdb/EMD-52282], EMD-52274 (soft-landed β-galactosidase without ice) [https://www.ebi.ac.uk/emdb/EMD-52274], EMD-52244 (soft-landed and rehydrated β-galactosidase (average)) [https://www.ebi.ac.uk/emdb/EMD-52244], EMD-52260 (soft-landed and rehydrated β-galactosidase (most similar)) [https://www.ebi.ac.uk/emdb/EMD-52260], EMD-52230 (plunge-frozen β-galactosidase) [https://www.ebi.ac.uk/emdb/EMD-52230], EMD-55805 (soft-landed and rehydrated β-galactosidase (most similar particles, as identified in a variability analysis without using the native particles as a reference)) [https://www.ebi.ac.uk/emdb/EMD-55805]. The corresponding data set are accessible on EMPIAR (EMPIAR-12558 [https://www.ebi.ac.uk/empiar/EMPIAR-12558], EMPIAR-12576, EMPIAR-12481, EMPIAR-12480, EMPIAR-12489, EMPIAR-12479, EMPIAR-12475). The atomic coordinates of the models have been deposited in the Protein Data Bank (PDB) under accession codes 9HKI (plunge-frozen β-galactosidase) [https://www.rcsb.org/structure/9HKI] and 9HLM (soft-landed and rehydrated β-galactosidase (most similar)) [https://www.rcsb.org/structure/9HLM].
