# Supplementary material for: Cryo-EM sample preparation with soft-landing and laser flash melting
Source: Nat Commun. 2026 Sep 15;17:10850. doi: 10.1038/s41467-026-77723-6 (PMC13672294; doi:10.1038/s41467-026-77723-6)
Supplement: Supplementary file 1 [file 41467_2026_77723_MOESM1_ESM.pdf]

Supplementary Information

Cryo-EM Sample Preparation with Soft-Landing and Laser Flash Melting

Sarah V. Barrass,[a] Tim K. Esser,[b†] Nathan J. Mowry,[a] Lukas Eriksson,[b,c] Jakub Wenz,[a‡]

Laurence T. Seeley,[b,c,d] Wyatt A. Curtis,[a] Lindsay A. Baker,[b,d] Marcel Drabbels,[a‡]

Stephan Rauschenbach,[b,c] Ulrich J. Lorenz[a*]

Affiliations:

[a] Ecole Polytechnique Fédérale de Lausanne (EPFL), Laboratory of Molecular Nanodynamics, CH-1015 Lausanne, Switzerland

[b] Kavli Institute for Nanoscience Discovery, Dorothy Crowfoot Hodgkin Building, University of Oxford, South Parks Road, Oxford OX1 3QU, UK

[c] Department of Chemistry, University of Oxford, Mansfield Road, Oxford OX1 3TA, UK

[d] Department of Biochemistry, University of Oxford, South Parks Road, Oxford OX1 3QU, UK

[†] Current affiliation: Thermo Fisher Scientific, De Schakel 2, 5651GH Eindhoven, Noord-Brabant, The Netherlands

[‡] Current affiliation: Ecole Polytechnique Fédérale de Lausanne (EPFL), Laboratory for Ultrafast X-ray Sciences, CH-1015 Lausanne, Switzerland

[*] To whom correspondence should be addressed. Email: ulrich.lorenz@epfl.ch

This PDF file includes:

A. Data collection

B. Single-particle reconstructions

C. Calculation of Fourier Shell Corelations

D. Variability analysis of soft-landed and rehydrated β-galactosidase

E. Model building

F. Supplementary figures

G. Supplementary tables

A. Data collection

Micrographs of soft-landed GroEL without and with an additional ice layer deposited on the sample (Fig. 2A,B) were collected at the COSMIC Cryo-EM Facility, University of Oxford, UK using a Talos Arctica 200 kV microscope equipped with a Falcon 4i detector (without ice) and with a Titan Krios G3 equipped with a GIF Bioquantum energy filter and a Gatan K3 camera (with ice). Thermo Fisher Scientific EPU v3.6 software was used to collect the data. All other datasets were collected at the Dubochet Center for Imaging in Lausanne, Switzerland, using a Titan Krios G4 microscope equipped with a Falcon 4i detector and a SelectrisX energy filter. Thermo Fisher Scientific EPU v3.10 software was used to collect the data. The data acquisition parameters for the different data sets are listed in Supplementary Tables 2 and 3.

B. Single-particle reconstructions

<u>Plunge-frozen GroEL</u>

Single-particle reconstructions were performed in CryoSPARC v4.5 [1]. The processing workflow is shown in Supplementary Figure 1. The micrographs were subjected to patch motion-correction, and movies with a total full-frame motion distance of more than 50 px were discarded. CTF estimation was performed using Patch CTF, and micrographs with an estimated resolution of worse than 6 Å, astigmatism greater than 1000 Å, or relative ice thickness values above 1.5 or below 0.9 were discarded. Particles were picked using a blob picker with a diameter of 120-180 Å, extracted with a box size of 512 px, and subjected to two rounds of 2D classification. The best classes were selected and used for template-based picking using a particle diameter of 150 Å. The particles were extracted with a box size of 512 px. After one round of 2D classifications, the selected particles were used for *ab-initio* reconstruction with two classes, resulting in one class representative of the intact protein (Supplementary Figure 1C, blue), and one junk class (Supplementary Figure 1C, grey). Following heterogeneous refinement, non-uniform refinement (D_7 symmetry) was performed on the intact protein class, with local and global CTF refinement and Ewald sphere correction, yielded a map at a resolution of 1.9 Å. The global and local resolution were estimated using gold-standard FSC thresholds of 0.143 and 0.5, respectively. The global B-factor was estimated from a linear fit of the Guinier plot between 10 Å and the resolution limit. The apical region of the map exhibits a lower resolution, which 3D classification (four classes) followed by non-uniform refinement of each class (C_7 symmetry), revealed

to be caused by the presence of a mixture of different states, which are shown in Supplementary Figure 11. The structures were visualized with UCSF ChimeraX 1.7 [2,3].

Soft-landed GroEL without ice

Single-particle reconstructions were performed in CryoSPARC 4.4.1 [1]. The processing workflow is shown in Supplementary Figure 2. The micrographs were subjected to patch motion-correction, and movies with a total full-frame motion distance of more than 50 px were discarded. CTF estimation was performed using Patch CTF and micrographs with a defocus tilt angle of greater than 15.5 degrees discarded. Particles were manually picked from one micrograph, extracted with a box size of 352 px, and subjected to three rounds of 2D classification. The best classes were selected and used for template-based picking using a particle diameter of 150 Å. The particles were extracted with a box size of 300 px, subjected to three rounds of 2D classification, followed by *ab-initio* reconstruction with one class. The particles were refined using heterogeneous refinement with three classes. Particles from classes two and three were then refined using non-uniform refinement with D_7 symmetry, against an initial model generated by low pass filtering the published cryo-EM map EMD-8750 to 30 Å. This yielded a map at a resolution of 6.7 Å. The global and local resolution were estimated using gold-standard FSC thresholds of 0.143 and 0.5, respectively. The global B-factor was estimated from a linear fit of the Guinier plot between 10 Å and the resolution limit. The structures were visualized with UCSF ChimeraX 1.7 [2,3].

Soft-landed GroEL with ice

Single-particle reconstructions were performed in CryoSPARC 4.4.1 [1]. The processing workflow is shown in Supplementary Figure 3. The micrographs were subjected to patch motion-correction. CTF estimation was performed using Patch CTF and micrographs with an estimated resolution of worse than 8.13 Å, average intensities above 507.24 or below -407.35, or relative ice thickness values above 1.19 were discarded. Particles were picked using template-based picking with templates generated from manual picking and a particle diameter of 140 Å. The particles were extracted with a box size of 352 px and subjected to three rounds of 2D classification. The best classes were selected for *ab-initio* reconstruction with five classes, followed by heterogeneous refinement with five classes. The best class was then refined with homogeneous refinement followed by non-uniform refinement (D_7 symmetry).

This yielded a map at a resolution of 4.8 Å. The global and local resolution were estimated using gold-standard FSC thresholds of 0.143 and 0.5, respectively. The global B-factor was estimated from a linear fit of the Guinier plot between 10 Å and the resolution limit. The structures were visualized with UCSF ChimeraX 1.7 [2,3].

Soft-landed and rehydrated GroEL

Single-particle reconstructions were performed in CryoSPARC v4.5 [1]. The processing workflow is shown in Supplementary Figure 4. Micrographs were collected from two grids. The micrographs were subjected to patch motion-correction, and movies with a total full-frame motion distance of more than 50 px were discarded. CTF estimation was performed using Patch CTF, and micrographs with an estimated resolution of worse than 6 Å, or relative ice thickness values above 1.2 or below 1.0 were discarded. In addition, micrographs identified as containing hexagonal ice, cubic ice or no ice by manual inspection were discarded. Templates were generated from the plunge-frozen GroEL reconstruction, low pass filtered to 20 Å and used for template-based picking using a particle diameter of 150 Å. The particles were extracted with a box size of 512 px. Particles extracted from grid one (1,722,594 particles) were subjected to two rounds of 2D classification, and the selected particles were used for *ab-initio* reconstruction, followed by non-uniform refinement (D_7 symmetry). Next, all template-picked particles from both grids were classified in 3D against the refined volume and ten decoy classes (random noise generated from the initial guess in an *ab-initio* reconstruction job) using heterogeneous refinement. Particles from the class corresponding to GroEL were then classified twice in 3D against the refined volume and five decoy classes. Finally, good particles were refined using non-uniform refinement (D_7 symmetry), yielded a map at a resolution of 3.9 Å. The global and local resolution were estimated using gold-standard FSC thresholds of 0.143 and 0.5, respectively. The global B-factor was estimated from a linear fit of the Guinier plot between 10 Å and the resolution limit. The structures were visualized with UCSF ChimeraX 1.7 [2,3].

Plunge-frozen β-galactosidase

Single-particle reconstructions were performed in CryoSPARC v4.5 [1]. The processing workflow is shown in Supplementary Figure 5. The micrographs were subjected to patch motion-correction, and movies with a total full-frame motion distance of more than 50 px were discarded. Contrast transfer function (CTF) estimation was performed using Patch CTF, and micrographs with an estimated resolution of worse than 6 Å, astigmatism greater than 1000 Å, or relative ice thickness values above 1.5 or below 0.9 were discarded. Particles were picked using a blob picker with a diameter of 80-180 Å, extracted with a box size of 512 px, and subjected to one round of 2D classification. The best classes were selected and used for template-based picking using a particle diameter of 180 Å. Following template picking, the particles were extracted with a box size of 600 px. After two rounds of 2D classifications, the selected particles were used for *ab-initio* reconstruction with two classes, resulting in one class representative of the intact tetrameric protein (Supplementary Figure 5C, blue), and one class of the dimeric protein (Supplementary Figure 5C, grey). Following heterogeneous refinement, and non-uniform refinement (C_1 symmetry) of the intact tetrameric protein, 3D classification was performed to remove damaged particles (Supplementary Figure 5C, grey). Finally, non-uniform refinement (D_2 symmetry) with local and global CTF refinement and Ewald sphere correction yielded a map at a resolution of 1.9 Å. The map was post processed using local filtering. The global and local resolution were estimated using gold-standard FSC thresholds of 0.143 and 0.5, respectively. The global B-factor was estimated from a linear fit of the Guinier plot between 10 Å and the resolution limit. The structures were visualized with UCSF ChimeraX 1.7 [2,3].

Soft-landed β-galactosidase without ice

Single-particle reconstructions were performed in CryoSPARC v4.5 [1]. The processing workflow is shown in Supplementary Figure 6. The micrographs were subjected to patch motion-correction, and CTF estimation was performed using Patch CTF. Particles were picked using a blob picker with a diameter of 80-200 Å, extracted with a box size of 540 px, and classified using two rounds of 2D classification. The best classes were selected, and an initial model generated using *ab-initio* reconstruction. The model was refined using non-uniform refinement with D_2 symmetry, yielded a map at a resolution of 5.9 Å. The map was post processed using local filtering. The global and local resolution were estimated using gold-standard FSC thresholds of 0.143 and 0.5, respectively. The global B-factor

was estimated from a linear fit of the Guinier plot between 10 Å and the resolution limit. The structures were visualized with UCSF ChimeraX 1.7 [2,3].

Soft-landed and rehydrated β-galactosidase (Average)

Single-particle reconstructions were performed in CryoSPARC v4.5 [1]. The processing workflow is shown in Supplementary Figure 7. Micrographs were collected from two grids and processed together. The micrographs were subjected to patch motion-correction, and movies with a total full-frame motion distance of more than 50 px were discarded. CTF estimation was performed using Patch CTF, and micrographs with an estimated resolution of worse than 6 Å, or relative ice thickness values above 1.2 or below 1.0 were discarded. In addition, micrographs identified as containing hexagonal ice, cubic ice or no ice by manual inspection were discarded. Particles were picked with a blob picker using a diameter of 80-180 Å, extracted with a box size of 512 px, and subjected to one round of 2D classification. The best classes were selected, and an initial model generated using *ab-initio* reconstruction, before being refined using non-uniform refinement with D_2 symmetry. The resulting volume was used to generate templates for template-based picking with a particle diameter of 180 Å. The particles were extracted with a box size of 512 px, subjected to two rounds of 2D classification, followed by *ab-initio* reconstruction with seven classes. The best class was then refined with non-uniform refinement (C_1 symmetry). Next, all template-picked particles were classified in 3D against the refined volume and five decoy classes (random noise generated from the initial guess in an *ab-initio* reconstruction job) using heterogeneous refinement, to improve particle classification. Finally, particles were subjected to one round of 2D classification and the good particles refined using non-uniform refinement (C_1 symmetry), yielded a map at a resolution of 2.9 Å. The global and local resolution were estimated using gold-standard FSC thresholds of 0.143 and 0.5, respectively. The global B-factor was estimated from a linear fit of the Guinier plot between 10 Å and the resolution limit. The structures were visualized with UCSF ChimeraX 1.7 [2,3].

C. Calculation of Fourier Shell Corelations

The map-to-map Fourier Shell Correlations (FSCs) for the reconstructions of soft-landed and rehydrated GroEL and ß-galactosidase were calculated against the corresponding native maps from plunge frozen samples. If required, the native map was resampled to have the same pixel size and box

size in ChimeraX. Both maps were then aligned in CryoSPARC v4.7.1 [1] with Align 3D Maps, and the FSC was calculated with the FSC (Validation) job, where a mask was generated for the maps of the soft-landed and rehydrated particles (threshold 0.2, dilation far 24 Å, dilation near 12 Å). The FSC values are reported at a threshold of 0.5. The local FSCs illustrated in Supplementary Figures 10 and 13 were calculated with the Local Resolution job.

For the reconstructions of β-galactosidase in Fig. 3A-E and Supplementary Figures 15, 16, and 18 we also calculated FSCs with the model of the native sample (Supplemetary Information I), which are listed in Supplementary Table 1 together with the map-to-map FSCs. The map-to-model FSCs were calculated as follows. A 2 Å map was generated from the model in ChimeraX, using the pixel and box size of the experimental map. Both maps were then aligned in CryoSPARC v4.7.1 with Align 3D Maps, and the FSC was calculated with the FSC (Validation) job, where a mask was generated for the maps of the soft-landed and rehydrated particles (threshold 0.2, dilation far 24 Å, dilation near 12 Å). Note that we cannot easily provide similar map-to-model FSCs for GroEL, since our sample appears to contain a mixture of different states (Supplementary Figure 11).

D. Variability analysis of soft-landed and rehydrated β-galactosidase

We used variability analysis to understand the motions that the particles undergo as they rehydrate during laser melting. Particles from the soft-landed and rehydrated β-galactosidase dataset (Fig. 3C) were combined with those of the native state (Fig. 3E), and 3D variability analysis in CryoSPARC was performed (filter resolution 10 Å, 3 variability components). Figure 3F shows the conformational distributions along the first component, which predominantly describes the expansion the particles undergo as they rehydrate. The distributions of the rehydrated and the native particles are well separated. This indicates that during flash melting, most particles have not quite approached a fully rehydrated conformation. Only the tail of the distribution overlaps with that of the native particles, suggesting that the particles at this end of the distribution have moved furthest along the trajectory associated with the rehydration. We selected 25,206 particles from this end of the distribution (as indicated in Fig. 3F) and performed an *ab-initio* reconstruction with one class and non-uniform

refinement (D_2 symmetry) to obtain the map in Fig. 3D, which has a resolution of 3.7 Å (workflow illustrated in Supplementary Figure 8).

In order to better understand the rehydration process, we subdivided the distribution of the rehydrated particles along the first component as indicated in Fig. 4D (subsets of even size, with 3 % of the particles left out at either end of the distribution) and generated reconstructions for each subset. A*b-initio* reconstruction with one class and non-uniform refinement (C_1 symmetry) yielded the volumes shown in Supplementary Figure 16. After aligning the maps, the Orientation diagnostics job in cryoSPARC was used to obtain the angular distributions of the particles (Fig. 4F–J, Supplementary Figure 16B,C).

We also tested how easily one can obtain a reconstruction of the rehydrated particles most closely resembling the native structure without including native particles in the variability analysis as a reference. The corresponding workflow is illustrated in Supplementary Figure 17. The particles of soft-landed and rehydrated β-galactosidase that give rise to the reconstruction in Fig. 3C were refined using non-uniform refinement with D_2 symmetry (Supplementary Figure 17A), and 3D variability analysis in CryoSPARC was performed (filter resolution 10 Å, 3 variability components). The third variability component, which is illustrated in Supplementary Figure 17B, predominantly describes an expansion of the particles. If we select 75,244 particles from the tail of the distribution (Supplementary Figure 17C) and perform an *ab-initio* reconstruction with one class (Supplementary Figure 17D), followed by non-uniform refinement (D_2 symmetry), we obtain the map in Supplementary Figure 17E with a resolution of 3.3 Å. As shown in Supplementary Figure 18, this map resembles the reconstruction in Fig. 3D of the rehydrated particles most similar to the native state, as identified in a variability analysis, where the native particles were included as a reference (Fig. 3F). However, the volume in Supplementary Figure 17E does not resemble the native structure as closely as the map in Fig. 3D, as indicated by a worse map-to-map FSC against the native (plunge frozen) map (6.9 Å for the volume in Supplementary Figure 17E compared to 4.5 Å for the map in Fig. 3D). Unsurprisingly, it is more difficult to identify the particles that most closely resemble the native structure from a variability analysis if the native particles are not included as a reference. This is also illustrated by Supplementary Figure 19, which shows the different particle distributions along the first principal component that we obtained in the joint variability analysis of Fig. 3F. The native particles are displayed in pink, and the rehydrated particles in purple. The

distribution of the most expanded particles that we selected in the variability analysis of Supplementary Figure 17B,C is shown in grey. It is closer to the native state than the distribution of all rehydrated particles, but contains a much broader range of particles than are included in the map of the most similar particles in Fig. 3D (box).

E. Model Building

A model of plunge-frozen β-galactosidase was obtained with the following procedure. An available β-galactosidase model (PDB: 6CVM) was rigidly fit into the locally filtered plunge-frozen β-galactosidase map using UCSF ChimeraX 1.7. The asymmetric unit within the model was then iteratively refined using the real space refine tool in PHENIX 1.21.1-5286-000 (Ref. [4]), with secondary structure and non-crystallographic symmetry constraints imposed, as well as the ISOLDE plugin in UCSF ChimeraX, and Coot [3,5,6].

A model for the soft-landed and revitrified (most similar) β-galactosidase map was obtained with a similar procedure. An available β-galactosidase model (PDB: 6CVM [7]) was rigidly fit into the sharpened map using UCSF ChimeraX 1.7. The asymmetric unit was then refined as above while imposing torsional and adaptive distance restraints derived from the starting structure in areas where the backbone density was poorly resolved. Imposing these restraints helped prevent unphysical geometries from occurring during the refinement in the areas of the map with a local resolution between 4 and 7 Å. Finally, the model was trimmed to remove areas where no density was observed. The quality of the models was evaluated using MolProbity [8] and PHENIX.

F. Supplementary Figures

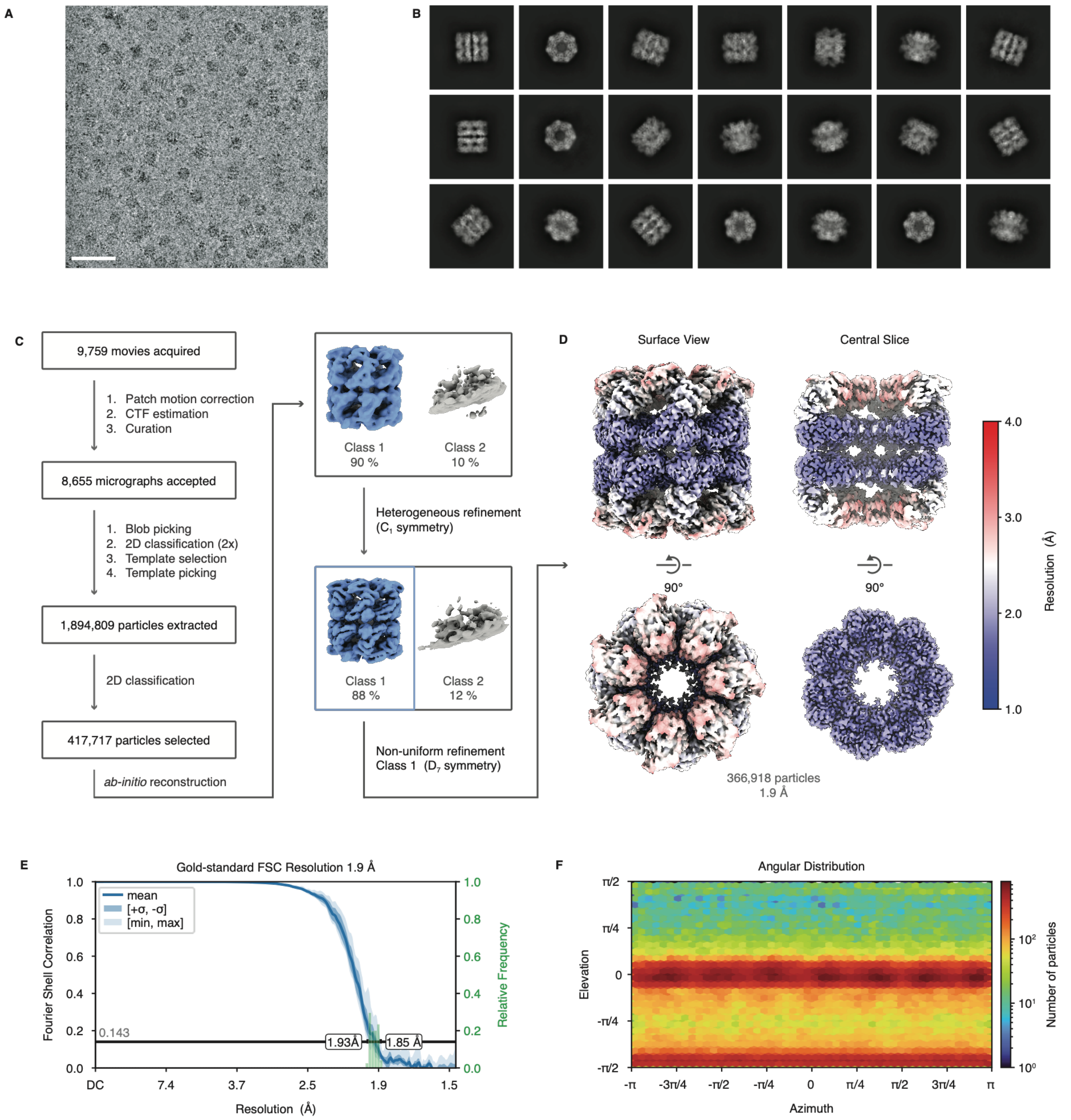

Supplementary Figure 1 | Cryo-EM reconstruction of plunge-frozen GroEL. (**A**) Representative micrograph, low pass filtered by 10 Å. Scale bar, 500 Å. (**B**) Selected 2D class averages of the dataset. (**C**) Single-particle reconstruction workflow in cryoSPARC, yielded a map at a resolution of 1.9 Å (**D**). (**D**) Final 3D reconstruction colored by local resolution. Note that different states of GroEL are present in our sample (Supplementary Figure 11), which lowers the resolution, particularly in the apical region. (**E**) Conical Gold-Standard Fourier Shell Correlation, with the black line indicating the 0.143 cut-off. The mean value is shown as a solid blue line, with the dark blue shading corresponding to one standard deviation, and the light blue shading indicating the minimum and maximum. A histogram of the

resolution values is shown in green; the minimum and maximum resolution are indicated. (**F**) Angular distribution of the particles.

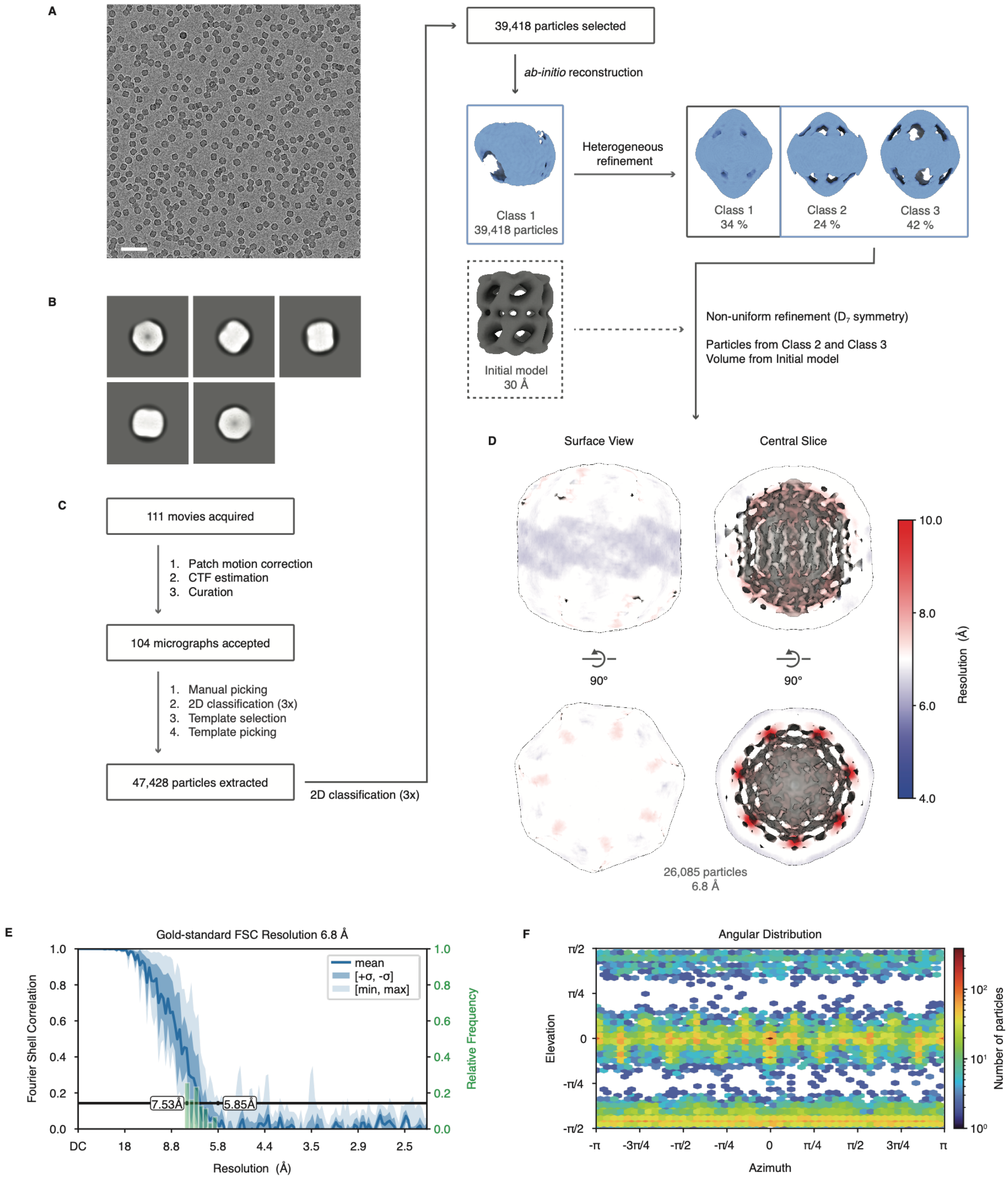

Supplementary Figure 2 | Cryo-EM reconstruction of soft-landed GroEL without ice. (**A**) Representative micrograph. Scale bar, 500 Å. (**B**) Selected 2D class averages of the dataset. (**C**) Single-particle reconstruction workflow in cryoSPARC, yielded a map at a resolution of 6.7 Å (**D**). (**D**) Final 3D reconstruction colored by local resolution. (**E**) Conical Gold-Standard Fourier Shell Correlation, with the black line indicating the 0.143 cut-off. The mean value is shown as a solid blue line, with the dark blue shading corresponding to one standard deviation, and the light blue shading

indicating the minimum and maximum. A histogram of the resolution values is shown in green; the minimum and maximum resolution are indicated. (**F**) Angular distribution of the particles.

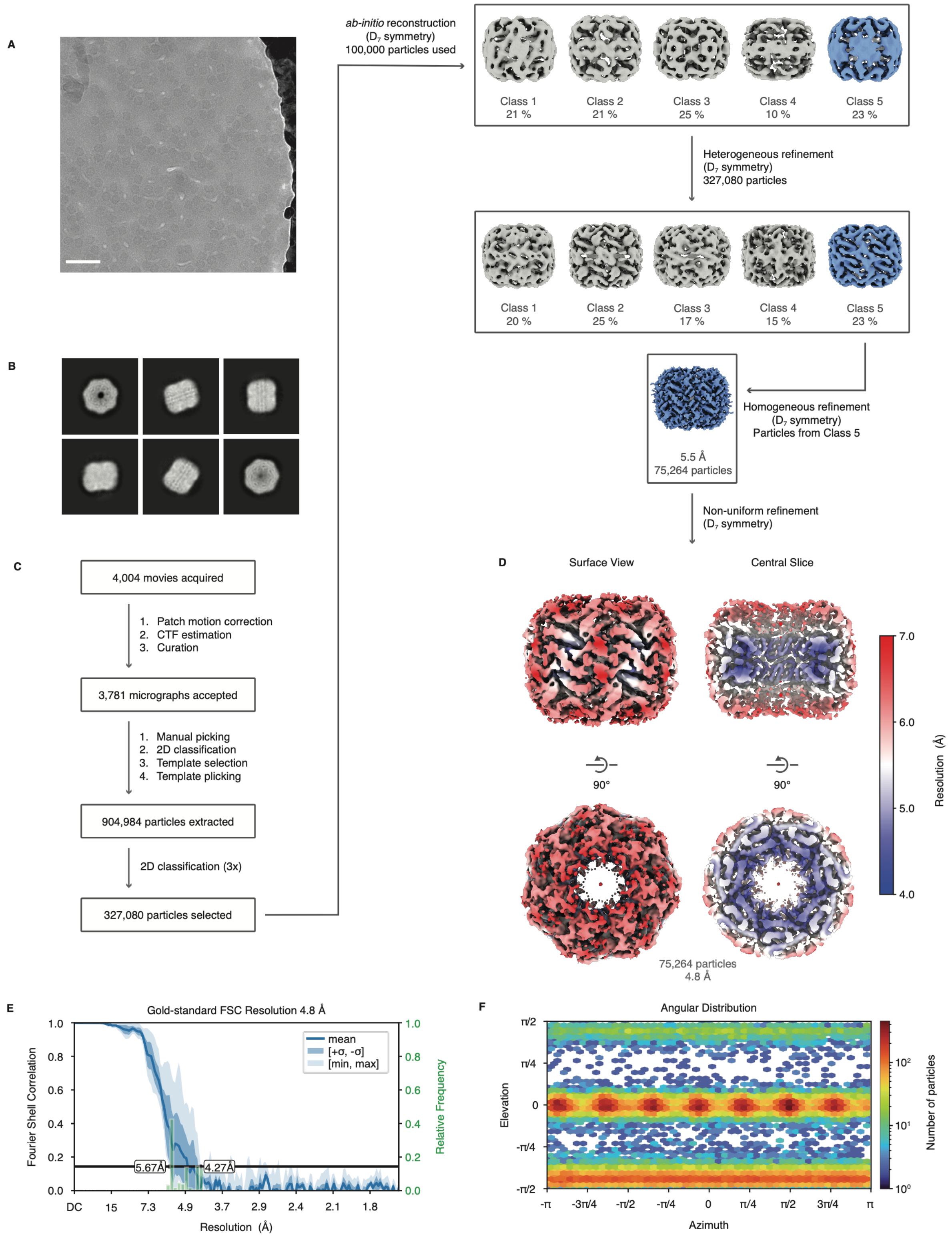

Supplementary Figure 3 | Cryo-EM reconstruction of soft-landed GroEL with ice. (**A**) Representative micrograph. Scale bar, 500 Å. (**B**) Selected 2D class averages of the dataset. (**C**) Single-particle reconstruction workflow in cryoSPARC, yielded a map at a resolution of 4.8 Å (**D**). (**D**) Final 3D reconstruction colored by local resolution. (**E**) Conical Gold-Standard Fourier Shell Correlation,

with the black line indicating the 0.143 cut-off. The mean value is shown as a solid blue line, with the dark blue shading corresponding to one standard deviation, and the light blue shading indicating the minimum and maximum. A histogram of the resolution values is shown in green; the minimum and maximum resolution are indicated. (**F**) Angular distribution of the particles.

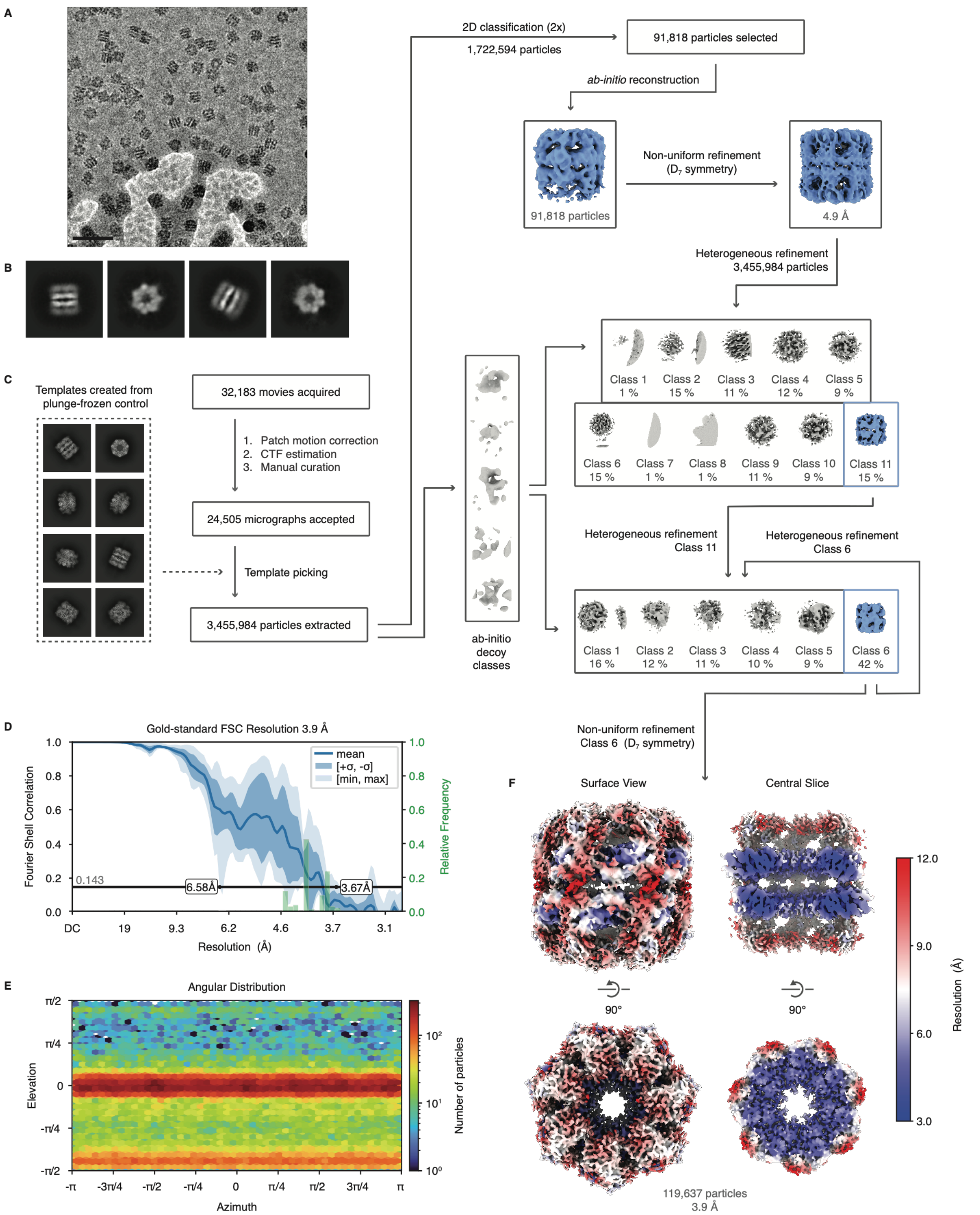

Supplementary Figure 4 | Cryo-EM reconstruction of soft-landed and rehydrated GroEL. (**A**) Representative micrograph, low pass filtered by 10 Å. Scale bar, 500 Å. (**B**) Selected 2D class averages of the dataset. (**C**) Single-particle reconstruction workflow in cryoSPARC, yielded a map at a resolution of 3.9 Å (**F**). (**D**) Conical Gold-Standard Fourier Shell Correlation, with the black line indicating the 0.143 cut-off. The mean value is shown as a solid blue line, with the dark blue shading

corresponding to one standard deviation, and the light blue shading indicating the minimum and maximum. A histogram of the resolution values is shown in green; the minimum and maximum resolution are indicated. (**E**) Angular distribution of the particles. (**F**) Final 3D reconstruction coloured by local resolution.

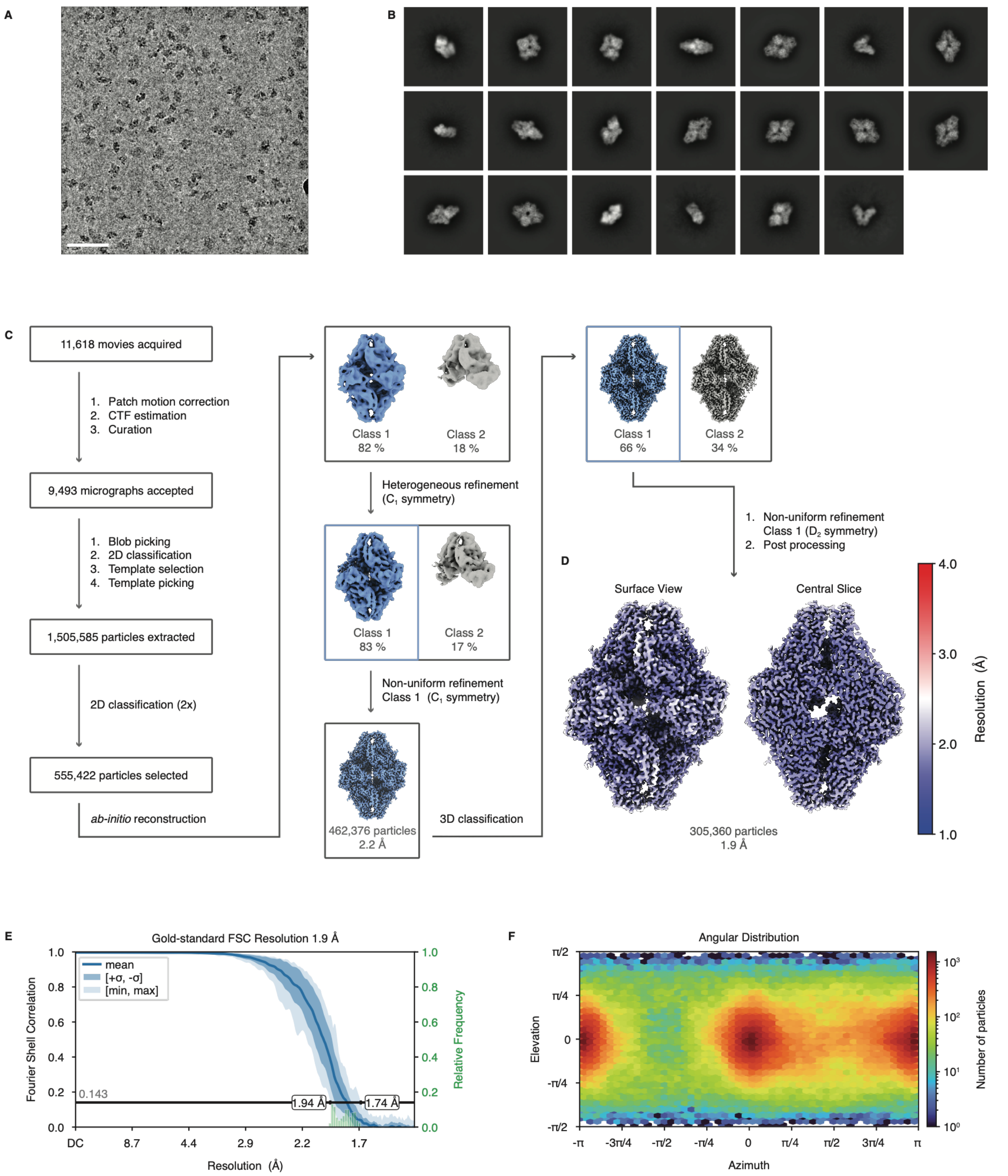

Supplementary Figure 5 | Cryo-EM reconstruction workflow for plunge-frozen β-galactosidase. (**A**) Representative micrograph, low pass filtered by 5 Å. Scale bar, 500 Å. (**B**) Selected 2D class averages of the dataset. (**C**) Single-particle reconstruction workflow in cryoSPARC. (**D**) Final 3D reconstruction with a resolution of 1.9 Å, colored by local resolution. (**E**) Conical Gold-Standard Fourier Shell Correlation, with the black line indicating the 0.143 cut-off. The mean value is shown as a solid blue line, with the dark blue shading corresponding to one standard deviation, and the light blue shading

indicating the minimum and maximum. A histogram of the resolution values is shown in green; the minimum and maximum resolution are indicated. (**F**) Angular distribution of the particles.

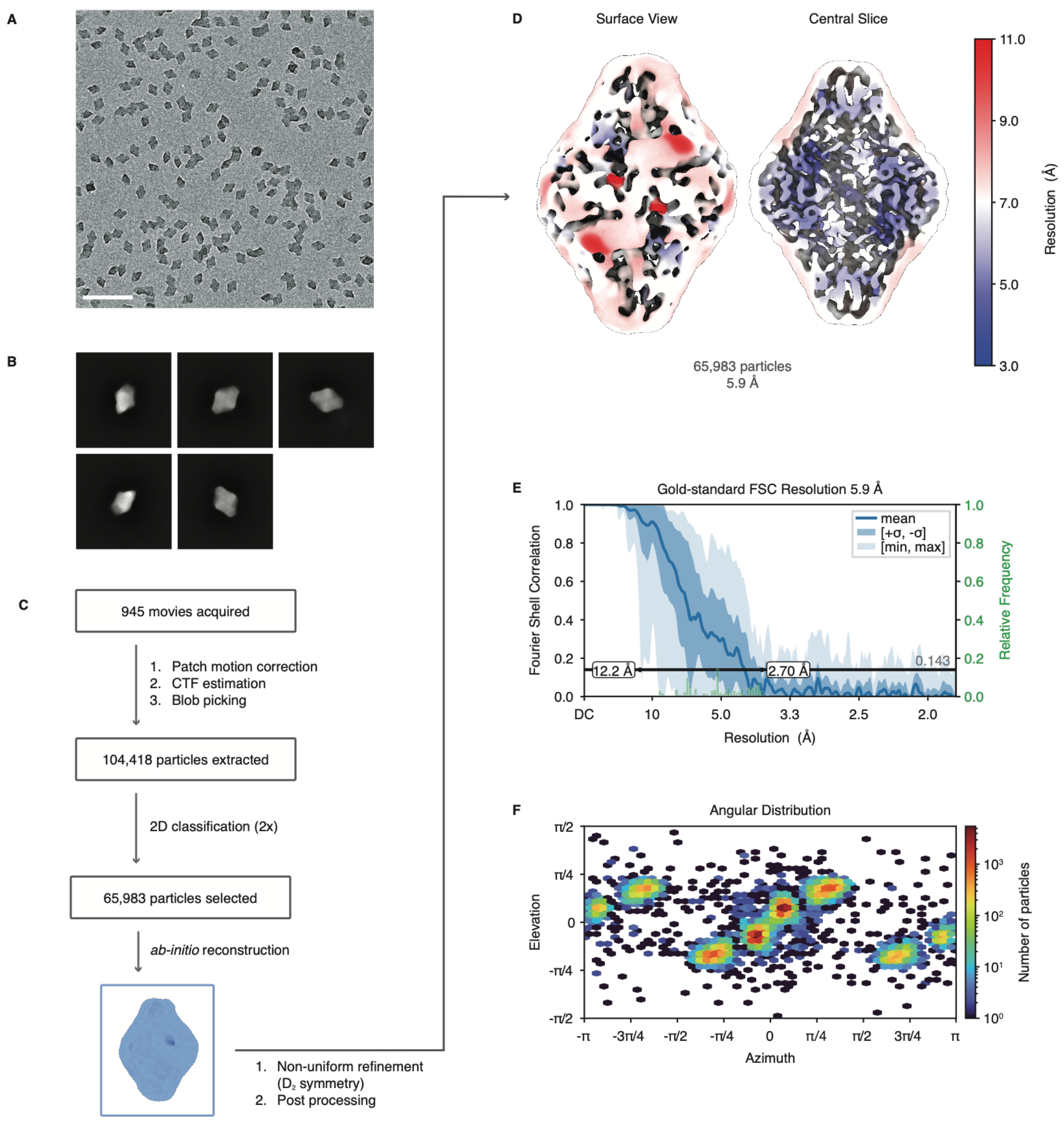

Supplementary Figure 6 | Cryo-EM reconstruction workflow for soft-landed β-galactosidase without ice. (**A**) Representative micrograph. Scale bar, 500 Å. (**B**) Selected 2D class averages of the dataset. (**C**) Single-particle reconstruction workflow in cryoSPARC, yielded a map at a resolution of 5.9 Å (**D**). (**D**) Final 3D reconstruction colored by local resolution. (**E**) Conical Gold-Standard Fourier Shell Correlation, with the black line indicating the 0.143 cut-off. The mean value is shown as a solid blue line, with the dark blue shading corresponding to one standard deviation, and the light blue shading indicating the minimum and maximum. A histogram of the resolution values is shown in green; the minimum and maximum resolution are indicated. (**F**) Angular distribution of the particles.

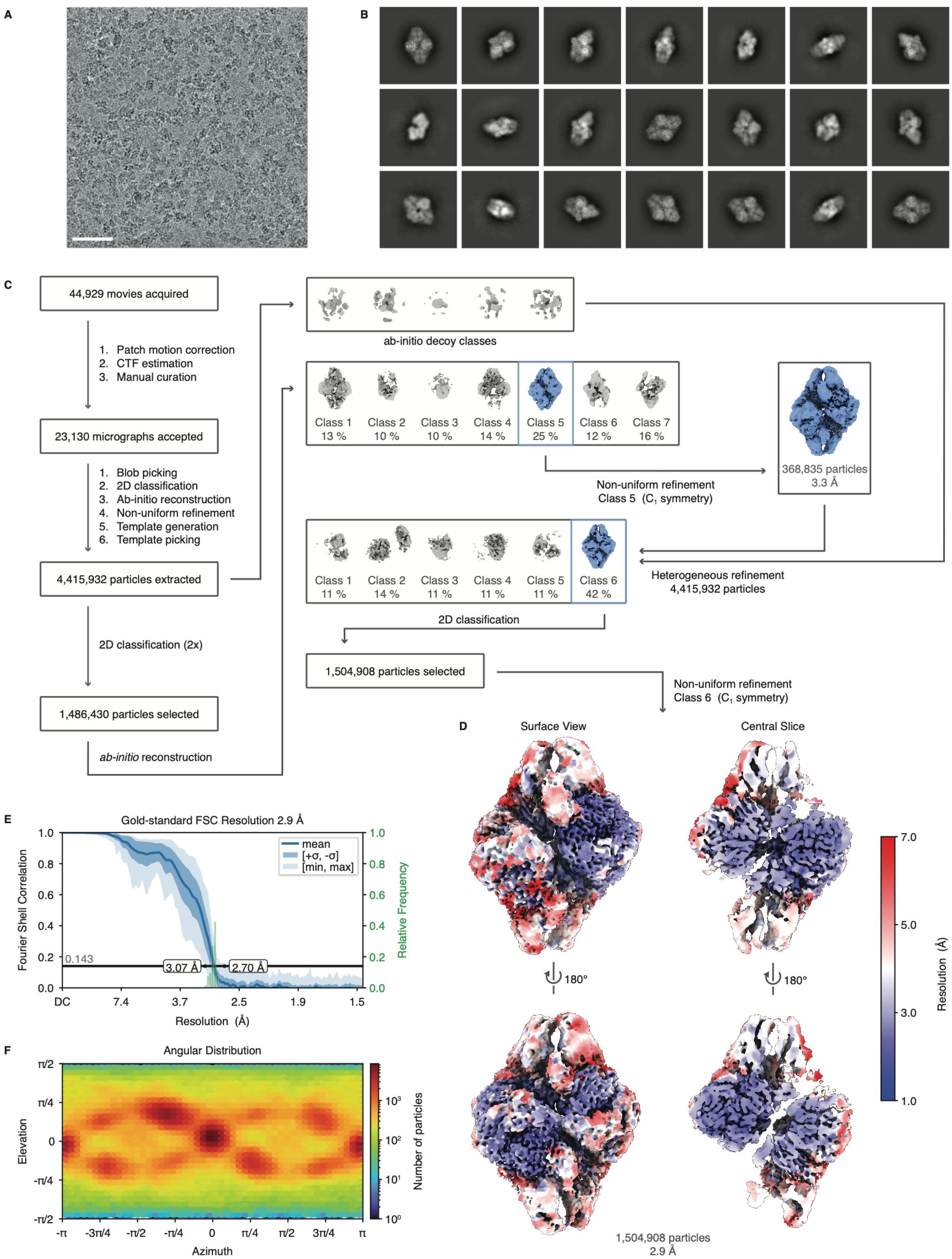

Supplementary Figure 7 | Cryo-EM reconstruction workflow for soft-landed and rehydrated β-galactosidase (Average). (**A**) Representative micrograph, low pass filtered by 5 Å. Scale bar, 500 Å. (**B**) Selected 2D class averages of the dataset. (**C**) Single-particle reconstruction workflow in cryoSPARC, yielded a map at a resolution of 2.9 Å (**D**). (**D**) Final 3D reconstruction colored by local

resolution. (**E**) Conical Gold-Standard Fourier Shell Correlation, with the black line indicating the 0.143 cut-off. The mean value is shown as a solid blue line, with the dark blue shading corresponding to one standard deviation, and the light blue shading indicating the minimum and maximum. A histogram of the resolution values is shown in green; the minimum and maximum resolution are indicated. (**F**) Angular distribution of the particles.

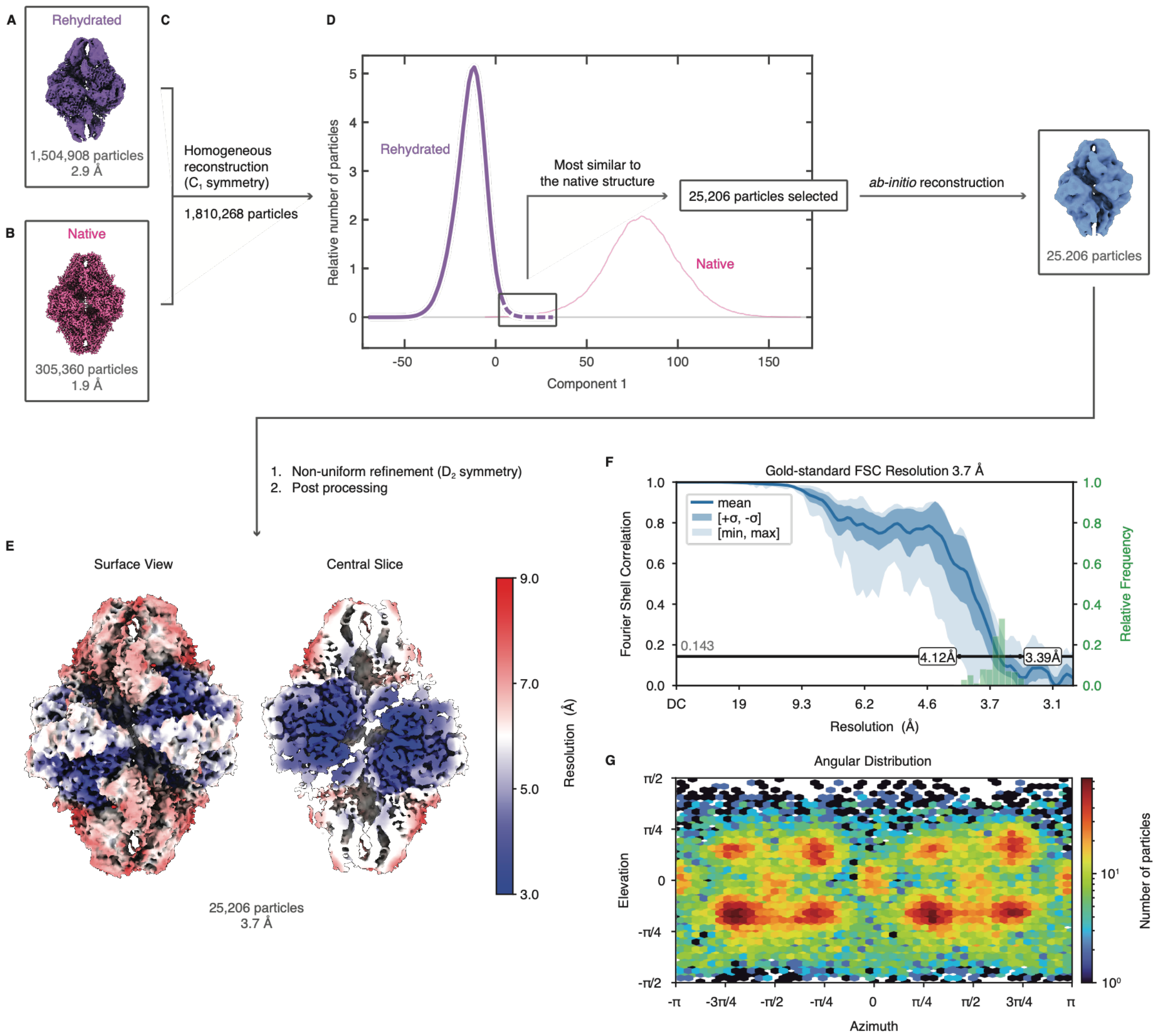

Supplementary Figure 8 | Cryo-EM reconstruction workflow for soft-landed and rehydrated β-galactosidase (most similar). (**A**) Reconstruction of soft-landed and rehydrated β-galactosidase (from Fig. 3C). (**B**) Reconstruction of native β-galactosidase from a plunge frozen sample (from Fig. 3E). (**C**) Particles from the reconstructions in (**A**) and (**B**) were combined and subjected to a 3D variability analysis in cryoSPARC. (**D**) Particle distributions along the first variability component, with the rehydrated and native particles shown in purple and pink, respectively. Rehydrated particles from the tail of the distribution (black box) were subjected to *ab-initio* reconstruction and refinement. (**E**) Reconstruction of the rehydrated particles from the tail of the distribution (from Fig. 3D). The map has a resolution of 3.7 Å; the local resolution is indicated in color. (**F**) Conical Gold-Standard Fourier Shell Correlation, with the black line indicating the 0.143 cut-off. The mean value is shown as a solid blue line, with the dark blue shading corresponding to one standard deviation, and the light blue shading

indicating the minimum and maximum. A histogram of the resolution values is shown in green; the minimum and maximum resolution are indicated. (**G**) Angular distribution of the particles.

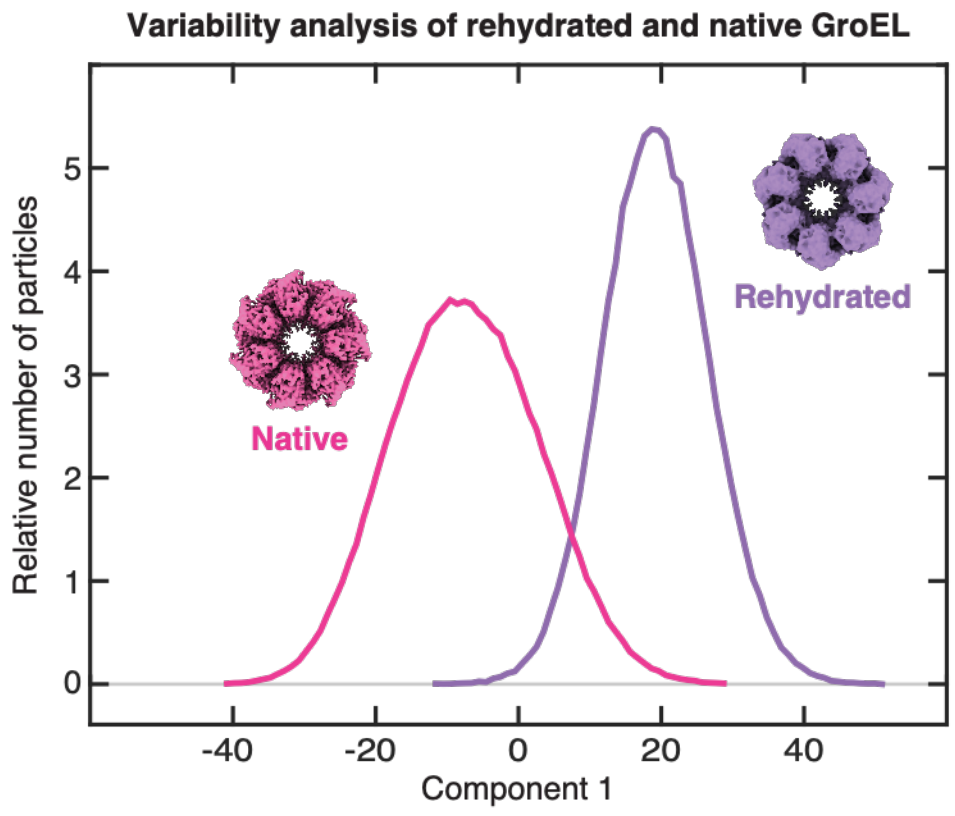

Supplementary Figure 9 | Variability analysis of the merged datasets of rehydrated and native GroEL. The particle distributions are shown as a function of the first variability component, which is mainly associated with the expansion of the particles as they rehydrate. The distributions of the rehydrated and native sample are shown in purple and pink, respectively. The variability analysis was performed in CryoSPARC (filter resolution 7 Å, 3 components).

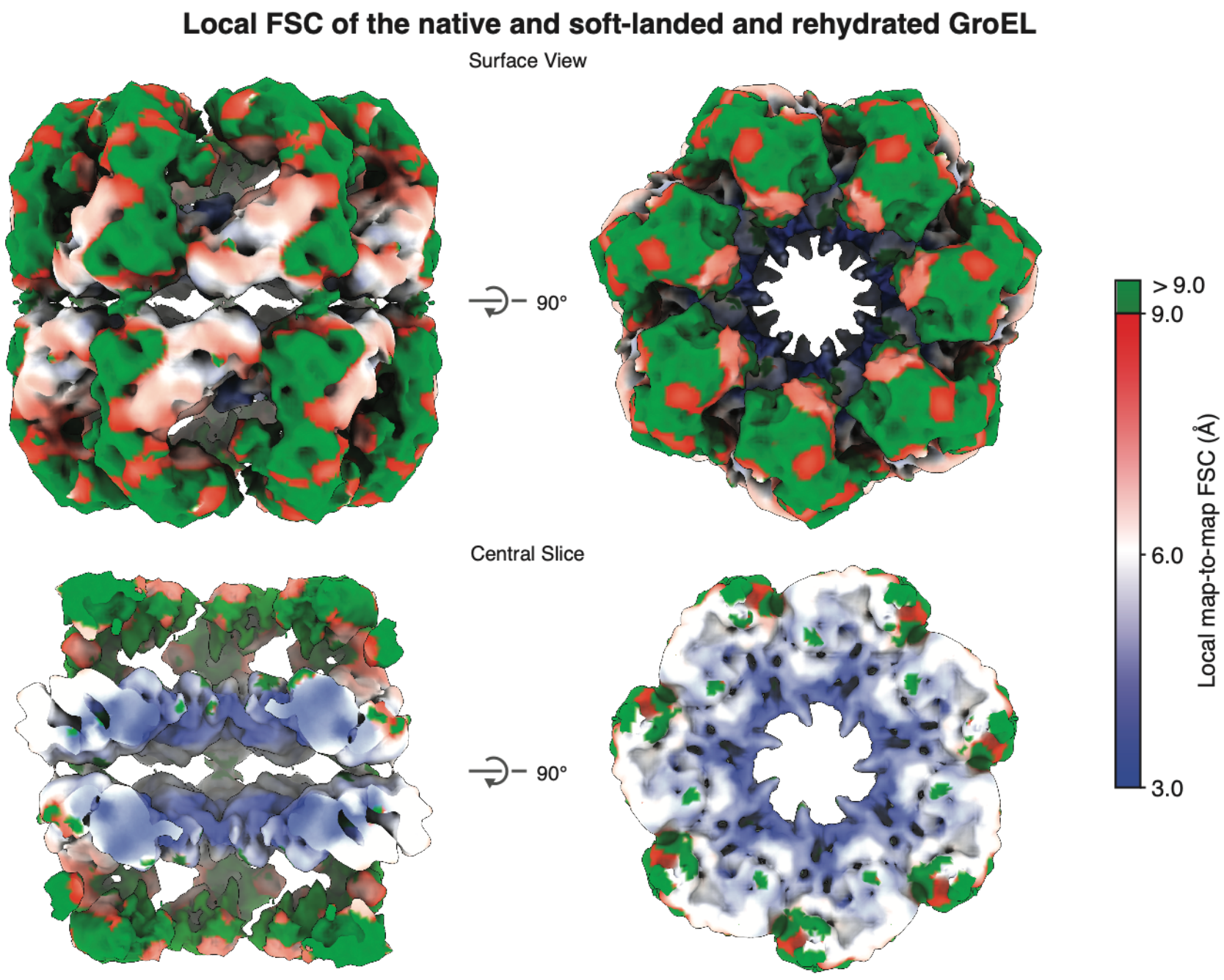

Supplementary Figure 10 | Local map-to-map FSC of rehydrated GroEL with the native map. The map of rehydrated GroEL from Fig. 2C is shown at a level of 4 σ, with the color overlay indicating the local map-to-map FSC value against the native map from Fig. 2D (threshold of 0.5). Regions with an FSC value of worse than 9 Å are shown in green.

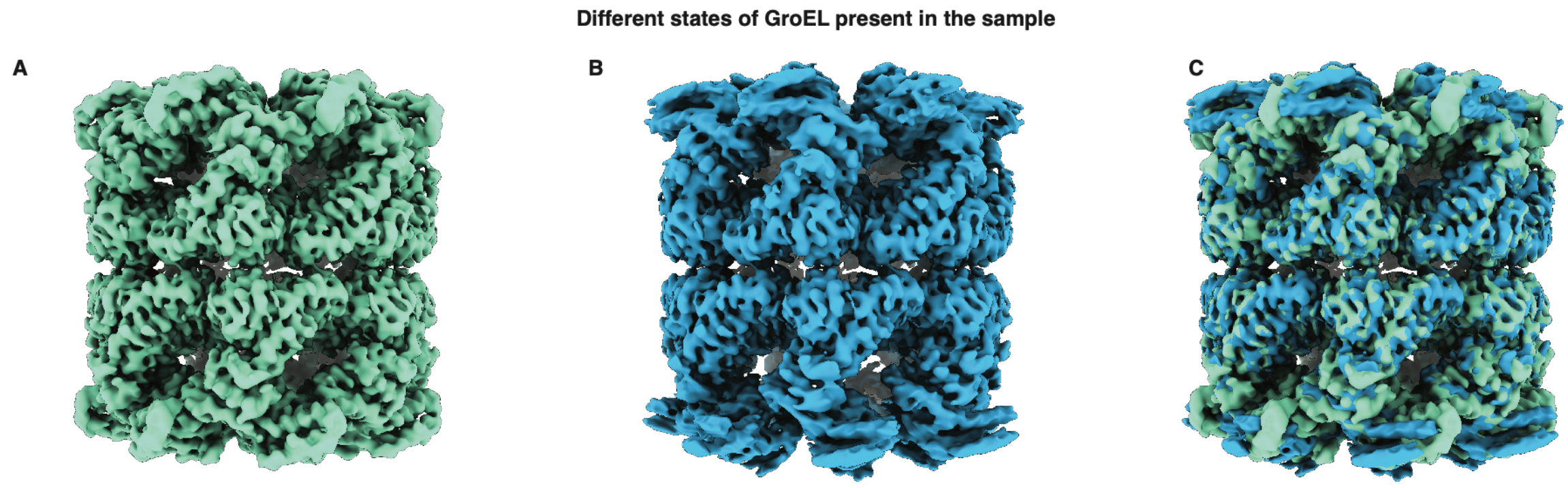

Supplementary Figure 11 | Comparison of different states of GroEL present in the native (plunge frozen) sample. (**A, B**) The two states. (**C**) Overlay.

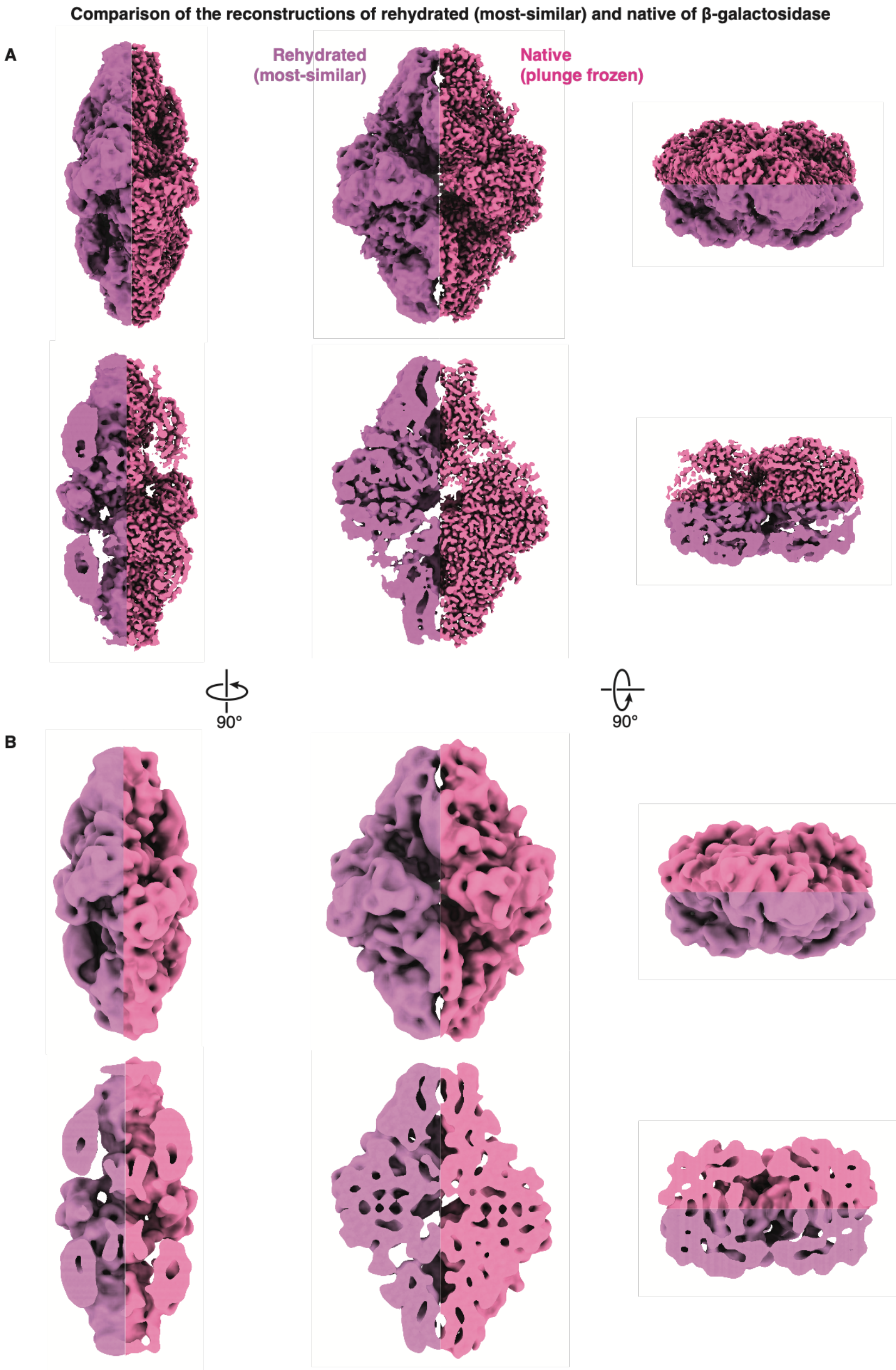

Supplementary Figure 12 | Comparison of the reconstructions of rehydrated (most similar) and native β-galactosidase. (**A**) The map of the rehydrated (most similar) particles from Fig. 3D is shown in purple, and the map of the native particles from Fig. 3E in pink. The maps are displayed at a threshold of 5 σ above the mean. (**B**) The same maps are low-pass filtered to 8 Å, and displayed at a threshold of 5 σ above the mean.

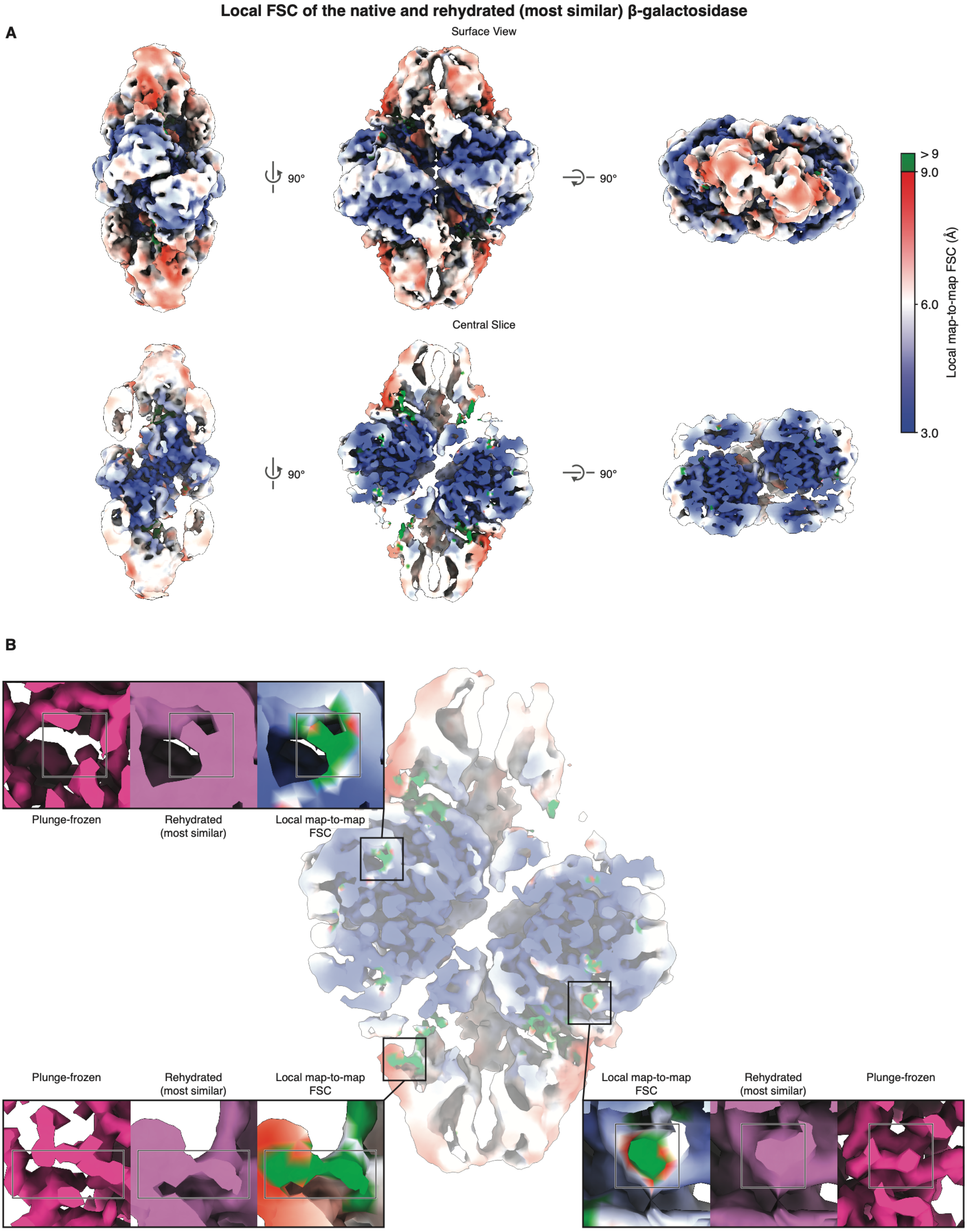

Supplementary Figure 13 | Local map-to-map FSC resolution of rehydrated ß-galactosidase (most similar) with the native map. A The map of rehydrated (most similar) ß-galactosidase from Fig. 3D is displayed at a level of 7 σ, with the color overlay indicating the local map-to-map FSC value against the native map from Fig. 3E (threshold of 0.5). Regions with an FSC value of worse than 9 Å

are shown in green. Some of the largest structural differences seem to occur around water-filled voids, suggesting that they have not yet fully re-expanded. **B** Details of such structural difference.

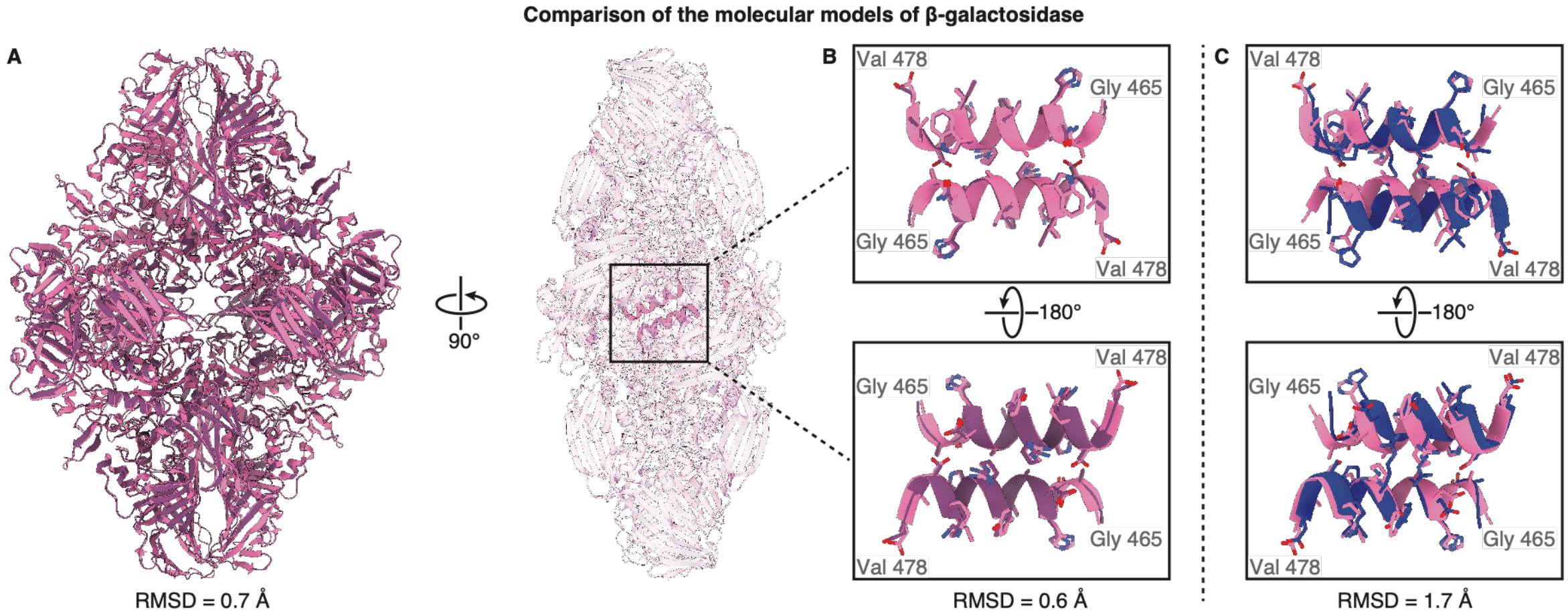

Supplementary Figure 14 | Comparison of the molecular models for soft-landed, rehydrated, and native β-galactosidase. (**A**) Comparison of the models for the rehydrated (most similar) and native structures (purple and pink, respectively). (**B**) Comparison of the subunit contacts within the well resolved core between the rehydrated (most similar) and native structures. The models closely resemble each other, with an RMSD of 0.6 Å. (**C**) Comparison of the same subunit contacts in the dehydrated (soft-landed with ice, PDB: 8Q7Y[9]) and native structures (blue and pink, respectively). The models show larger differences with an RMSD of 1.7 Å.

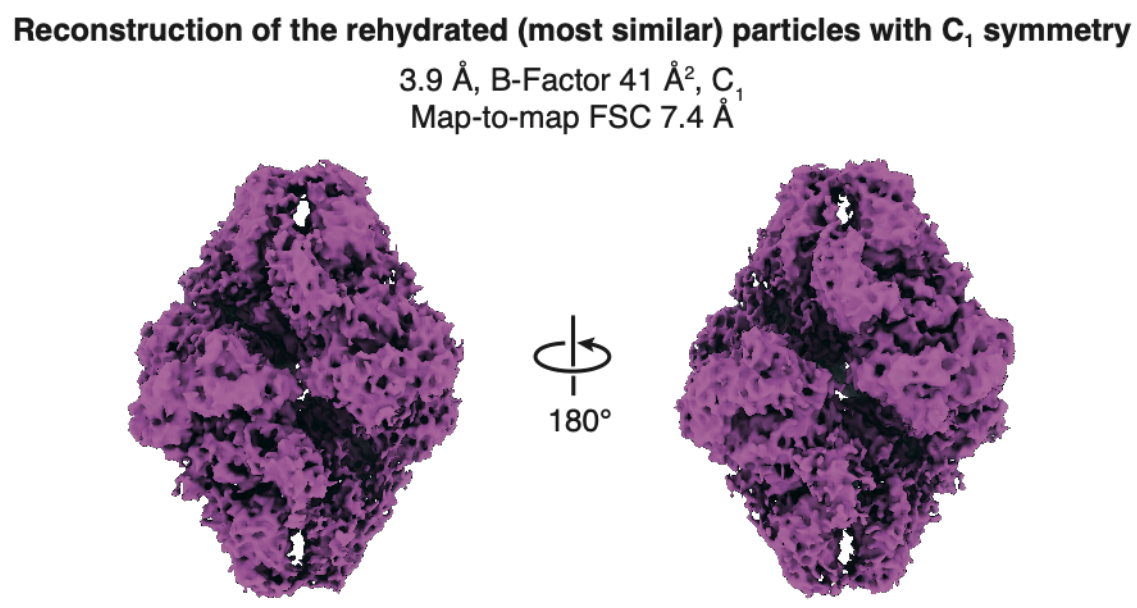

Supplementary Figure 15 | Reconstruction of the rehydrated (most similar) particles with C_1 symmetry. Both sides of the particles have expanded symmetrically. The map is shown at a threshold of 5 σ above the mean.

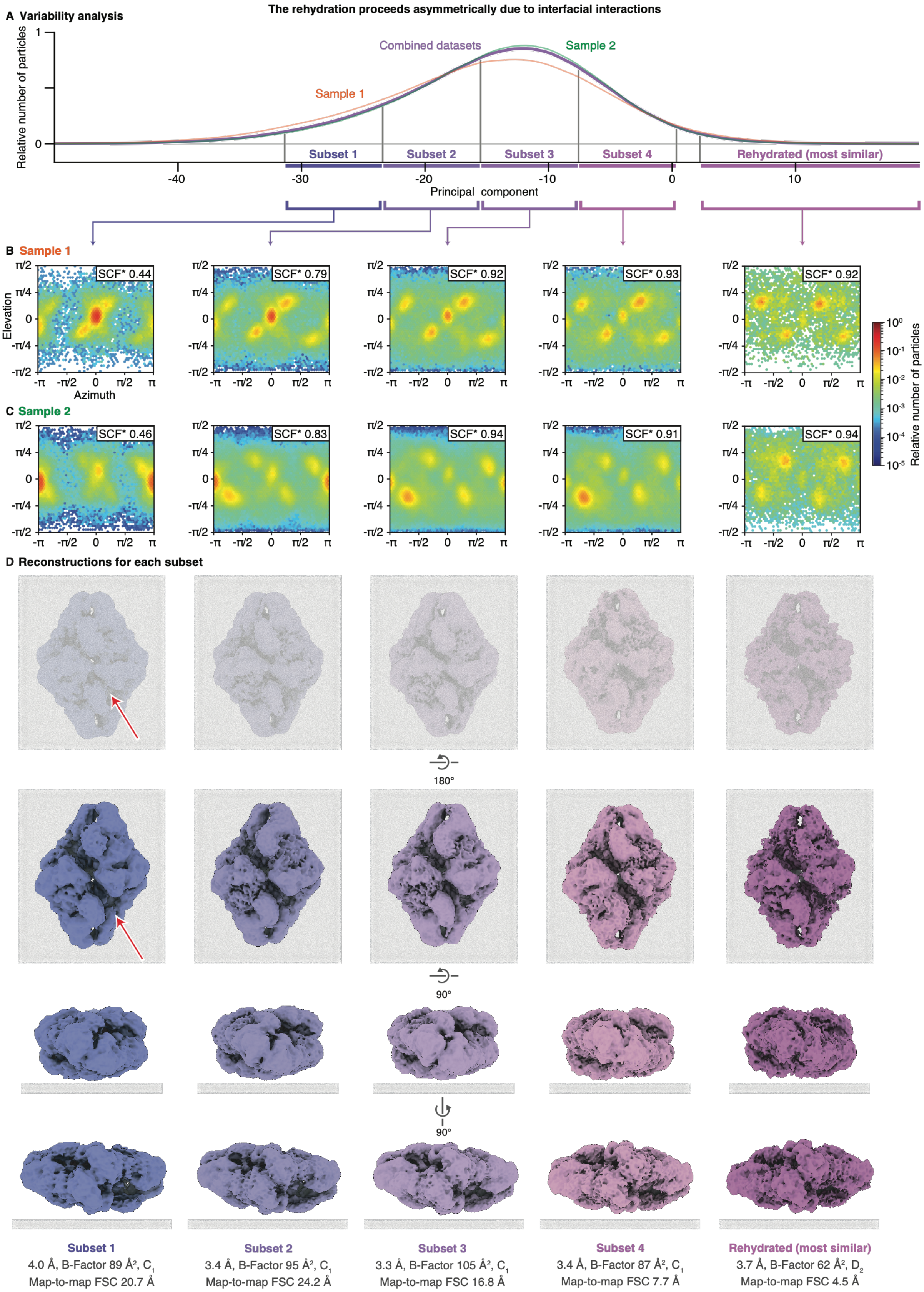

Supplementary Figure 16 | The rehydration proceeds asymmetrically due to interfacial interactions. (**A**) Distribution of the rehydrated β-galactosidase particles along the first variability

component for the entire dataset collected from two different samples (purple, from Fig. 4D), as well as for each of the samples (orange and green). The central portion of the distribution is subdivided into 4 subsets. The subset of particles most similar to the native state that are included in the reconstruction in Fig. 3D is also indicated. (**B**) Angular distributions of the particles in each subset for the first sample, with the corresponding SCF* values indicated (from Fig. 4F–I). Along the principal component, preferred orientation decreases. (**C**) Angular distributions for the second sample. During imaging, this sample was apparently mounted with the opposite side facing up compared to the first sample, which shifts the angular distribution by 180 degrees. This can for example be seen for the most asymmetric particles (subset 1), whose preferred orientation (adsorption of the particles with one of their faces) is rotated by 180 degrees. Note that besides this rotation, the angular distributions for sample 2 also show other changes to the preferred orientations of the particles, which is likely the result of small differences in the interfacial properties of the two samples. (**D**) Reconstructions for each subset for the combined particles of both samples. The map of the first subset shows significant asymmetry, with one face of the protein more expanded than the other. Arrows highlight the asymmetry in the subunit contacts on both faces. As preferred orientation decreases along the principal component, the volumes become more symmetric. This suggests that the interfacial interactions that lead to preferred orientation also hinder the rehydration of the particles, causing them to become asymmetric. The putative interaction with the interface is schematically indicated (grey). All reconstructions are performed with C_1 symmetry. The maps are displayed with thresholds of 5 σ above the mean. The resolution, B-factor, and symmetry are indicated, as well as the map-to-map FSC (Supplementary Information C, threshold 0.5). Note that for the compacted structures, the map-to-map FSC is not meaningful.

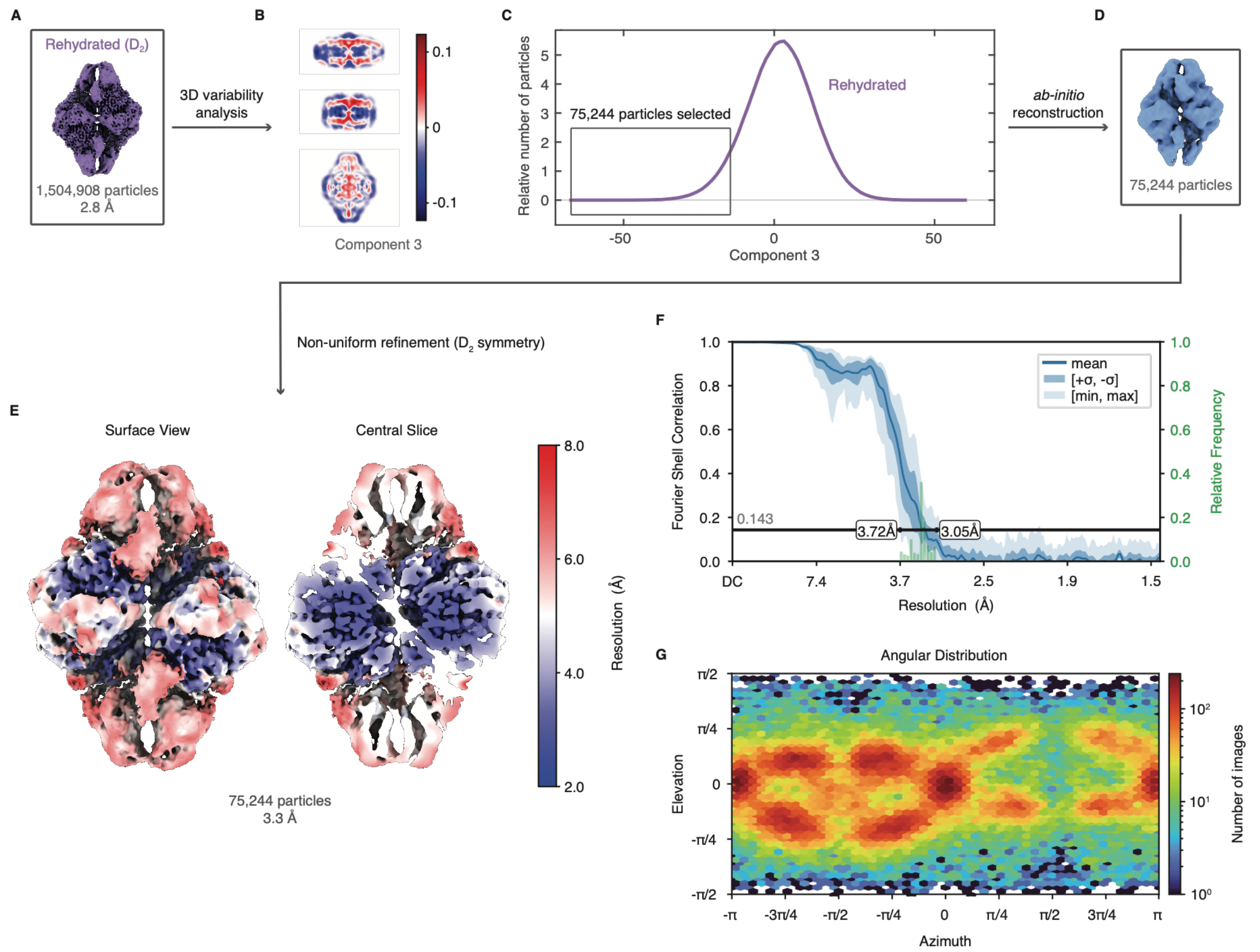

Supplementary Figure 17 | Cryo-EM reconstruction workflow for soft-landed and rehydrated β-galactosidase — selection of the particles most closely resembling the native structure from a variability analysis that does not include native particles as a reference. (**A**) Reconstruction of soft-landed and rehydrated β-galactosidase after non-uniform refinement with D_2 symmetry. (**B**) The particles were subjected to a 3D variability analysis in cryoSPARC without including native particles as a reference (filter resolution 10 Å, 3 variability components). The third variability component is illustrated, which predominantly describes an expansion of the particles. (**C**) Particle distribution along the third variability component. (**D**) Particles from the tail of the distribution in (**C**), marked with a black box, were subjected to ab-initio reconstruction. (**E**) Non-uniform refinement with D_2 symmetry yielded a map with 3.3 Å resolution; the local resolution is indicated in color. (**F**) Conical Gold-Standard Fourier Shell Correlation, with the black line indicating the 0.143 cut-off. The mean value is shown as a solid blue line, with the dark blue shading corresponding to one standard deviation, and the light blue shading indicating the minimum and maximum. A histogram of the resolution values is shown in green; the minimum and maximum resolution are indicated. (**G**) Angular distribution of the particles.

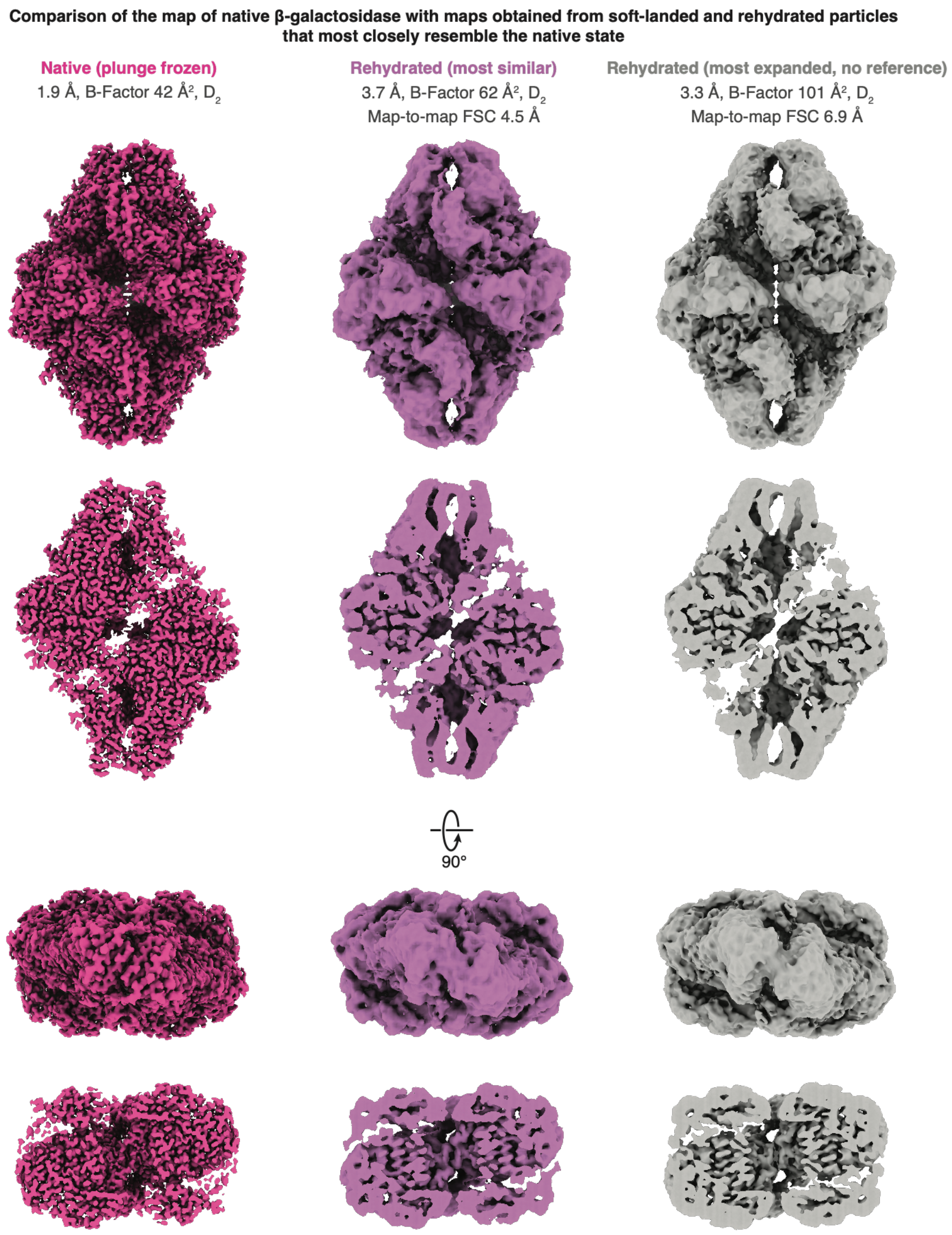

Supplementary Figure 18 | Comparison of the map of native β-galactosidase with maps obtained from soft-landed and rehydrated particles that most closely resemble the native state, as determined from a variability analysis. The native structure is shown on the left (from Fig. 3E). The volume in the middle (from Fig. 3D) represents a reconstruction of the rehydrated particles that most closely resemble the native state, as determined from the variability analysis in Fig. 3F, which includes native particles. The map on the right (from Supplementary Figure 17E) corresponds to the reconstruction of the particles that are most expanded, as determined from the variability analysis in Supplementary Figure 17B,C, which was carried out without including native particles as a reference.

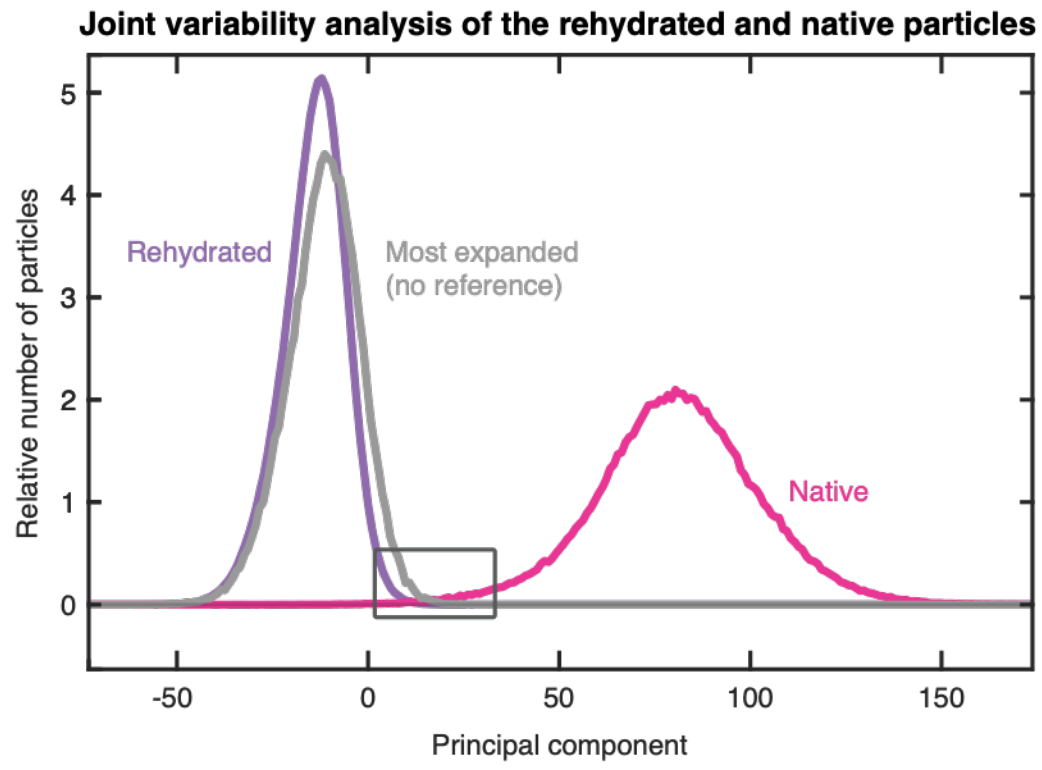

Supplementary Figure 19 | Joint variability analysis of the rehydrated and native particles (from Fig. 3F), with the distribution of the particles indicated that are most expanded, as determined from the variability analysis in Supplementary Figure 17B,C. The particle distribution is shown along the first principal component with the rehydrated particles in purple and the native particles in pink. A box marks the subset of rehydrated particles that were used for the reconstruction in Fig. 3D. The most expanded particles selected in the variability analysis of Supplementary Figure 17B,C (no native particles included as a reference) are shown in grey. Their distribution is shifted towards the native state compared with the distribution of all rehydrated particles.

G. Supplementary Tables

Supplementary Table 1 | Comparison of the map-to-map and map-to-model Fourier Shell Correlation (FSC) values of ß-galactosidase at a threshold of 0.5.

Experimental map	Map-to-map FSC (Å)	Map-to-model FSC (Å)	Figure
Plunge-frozen		2.0	Fig. 3
Soft-landed without ice	29.0	43.8	Fig. 3
Soft-landed with ice	42.5	41.8	Fig. 3
Rehydrated (average)	13.9	19.1	Fig. 3
Rehydrated (most similar)	4.5	7.6	Fig. 3
Rehydrated (most similar, C_1 symmetry)	7.4	8.9	Suppl. Fig. 15
3DVA subset 1	20.7	20.3	Suppl. Fig. 16
3DVA subset 2	24.2	20.0	Suppl. Fig. 16
3DVA subset 3	16.8	19.8	Suppl. Fig. 16
3DVA subset 4	7.7	12.4	Suppl. Fig. 16
Rehydrated (most similar, no reference)	6.9	9.0	Suppl. Fig. 18

Supplementary Table 2 | Data collection parameters as well as reconstruction and modelling statistics for GroEL.

Figure	Fig 2D	Fig 2A	Fig 2B	Fig 2C
**Name**	**Plunge-frozen**	**Soft-landed without ice**	**Soft-landed with ice**	**Soft-landed and rehydrated**
EMDB accession	EMD-52282	EMD-52627	EMD-52626	EMD-52284
**Data collection**				
Microscope	Thermo Fisher Titan Krios G4	Thermo Fisher Talos Arctica	Thermo Fisher Titan Krios G3	Thermo Fisher Titan Krios G4
Detector	Falcon 4i	Falcon 4i	Gatan K3	Falcon 4i
Energy Filter	SelectrisX	-	GIF Bioquantum	SelectrixX
Energy Slit	10 eV	-	20 eV	10 eV
Voltage (kV)	300	200	300	300
Electron exposure (e^{-}/Å^2)	60	40	40	60
Magnification	165,000	105,000	105,000	165,000
Defocus range (µm)	-0.8 – -2.2	-1.0 – -2.25	-1.0 – -2.5	-0.8 – -2.2
Pixel size (Å)	0.726	1.17	0.83	0.726
**Data processing**				
Initial movies (no.)	9,759	111	4,004	44,000
Symmetry imposed	D_7	D_7	D_7	D_7
Initial particle images (no.)	1,894,809	47,428	904,984	3,455,984
Final particle images (no.)	366,916	26,085	75,264	119,637
Map resolution (Å)	1.9	6.7	4.8	3.9
FSC threshold	0.143	0.143	0.143	0.143
Map resolution range (Å)	1.6 – 4.0	6.2 – 10	4.5 - 9.4	3.5 – 12.0

Supplementary Table 3 | Data collection parameters as well as reconstruction and modelling statistics for β-galactosidase.

Figure	Fig 3E	Fig 3A	Fig 3C	Fig 3D	Suppl. Fig. 18
**Name**	**Plunge-frozen**	**Soft-landed without ice**	**Soft-landed and rehydrated (Average)**	**Soft-landed and rehydrated (most similar)**	**Soft-landed and rehydrated (most expanded, no reference)**
EMDB accession	EMD-52230	EMD-52274	EMD-52244	EMD-52260	EMD-55805
PDB accession	9HKI			9HLM	
**Data collection**					
Microscope			Thermo Fisher Titan Krios G4		
Detector			Falcon 4i		
Energy Filter			SelectrisX		
Energy Slit			10 eV		
Voltage (kV)			300		
Electron exposure ($e^{-}/Å^2$)			60		
Magnification	165,000	130,000	165,000	165,000	165,000
Defocus range (μm)	-0.8 – -2.2				
Pixel size (Å)	0.726	0.926	0.726	0.726	0.726
**Data processing**					
Initial movies (no.)	11,618	945	44,929	44,929	44,929
Symmetry imposed	D_2	D_2	C_1	D_2	D_2
Initial particle images (no.)	1,505,585	104,418	4,415,932	4,415,932	4,415,932
Final particle images (no.)	305,360	65,983	1,504,908	25,206	75,244
Map resolution (Å)	1.9	5.9	2.9	3.7	3.3
FSC threshold	0.143	0.143	0.143	0.143	0.143
Map resolution range (Å)	1.6 – 3.5	4.0 – 12.6	1.6 – 5.4	3.2 – 8.0	2.8 – 7.0
**Refinement**		No atomic model built	No atomic model built		No atomic model built
Initial model used (PDB ID)	6CVM	-	-	6CVM	-
Model resolution (Å)	1.8	-	-	3.8	-
FSC threshold	0.143	-	-	0.143	-
Model resolution range (Å)	1.8/1.8/1.9	-	-	3.6/3.8/3.9	-
FSC threshold	0/0.143/0.5	-	-	0/0.143/0.5	-
Map sharpening *B* factor ($Å^2$)	41.9	-	-	62.7	-
Model composition					
Non-hydrogen atoms	36752	-	-	32040	-

Protein residues	4084	-	-	3984	-
Waters	3952	-	-	-	-
Ligands	12	-	-	-	-
Average *B* factors (Å^2)					
Protein	10.02	-	-	96.18	-
Ligand	8.52	-	-	-	-
Water	10.80			-	-
R.m.s. deviations					
Bond lengths (Å)	0.004	-	-	0.005	-
Bond angles (°)	1.036	-	-	1.026	-
Validation					
MolProbity score	1.08	-	-	2.17	-
Clashscore	2.74	-	-	21.26	-
Poor rotamers (%)	0.34	-	-	0.23	-
Ramachandran plot					
Favored (%)	97.94	-	-	95.03	-
Allowed (%)	2.06	-	-	4.67	-
Disallowed (%)	0.00	-	-	0.30	-

Supplementary References

1. Punjani, A., Rubinstein, J. L., Fleet, D. J. & Brubaker, M. A. cryoSPARC: algorithms for rapid unsupervised cryo-EM structure determination. *Nat Methods* **14**, 290–296 (2017).
2. Pettersen, E. F. *et al.* UCSF CHIMERAX : Structure visualization for researchers, educators, and developers. *Protein Science* **30**, 70–82 (2021).
3. Goddard, T. D. *et al.* UCSF ChimeraX: Meeting modern challenges in visualization and analysis: UCSF ChimeraX Visualization System. *Protein Science* **27**, 14–25 (2018).
4. Liebschner, D. *et al.* Macromolecular structure determination using X-rays, neutrons and electrons: recent developments in Phenix. *Acta Cryst D* **75**, 861–877 (2019).
5. Croll, T. I. ISOLDE: a physically realistic environment for model building into low-resolution electron-density maps. *Acta Cryst D* **74**, 519–530 (2018).
6. Emsley, P., Lohkamp, B., Scott, W. G. & Cowtan, K. Features and development of Coot. *Acta Cryst D* **66**, 486–501 (2010).
7. Bartesaghi, A. *et al.* Atomic Resolution Cryo-EM Structure of β-Galactosidase. *Structure* **26**, 848-856.e3 (2018).
8. Williams, C. J. *et al.* MolProbity: More and better reference data for improved all-atom structure validation. *Protein Science* **27**, 293–315 (2018).
9. Esser, T. K. *et al.* Cryo-EM of soft-landed β-galactosidase: Gas-phase and native structures are remarkably similar. *Science Advances* **10**, eadl4628 (2024).
