# Supplementary material for: Cryo-EM sample preparation with soft-landing and laser flash melting
Source: Nat Commun. 2026 Sep 15;17:10850. doi: 10.1038/s41467-026-77723-6 (PMC13672294; doi:10.1038/s41467-026-77723-6)
Supplement: Supplementary file 2 [file 41467_2026_77723_MOESM2_ESM.pdf]

Description of Additional Supplementary Files

File Name: Supplementary Movie 1

Description: Movie illustrating the structural changes of the rehydrated particles along the first principal component of the variability analysis of Fig. 3F. The movie represents a morph between the reconstructions obtained for the four subsets and the most similar map in Fig. S16, with all volumes displayed at a contour level of 5 σ. Four views of the maps are provided, which are arranged as in Fig. S16.
