# Supplementary material for: Cryo-EM sample preparation with soft-landing and laser flash melting
Source: Nat Commun. 2026 Sep 15;17:10850. doi: 10.1038/s41467-026-77723-6 (PMC13672294; doi:10.1038/s41467-026-77723-6)
Supplement: Supplementary file 3 [file 41467_2026_77723_MOESM4_ESM.pdf]

nature portfolio

Corresponding author(s): Ulrich J. Lorenz

Last updated by author(s): 22.7.2026

Reporting Summary

Nature Portfolio wishes to improve the reproducibility of the work that we publish. This form provides structure for consistency and transparency in reporting. For further information on Nature Portfolio policies, see our Editorial Policies and the Editorial Policy Checklist.

Statistics

For all statistical analyses, confirm that the following items are present in the figure legend, table legend, main text, or Methods section.

n/a	Confirmed	
☐	☒	The exact sample size (*n*) for each experimental group/condition, given as a discrete number and unit of measurement
☒	☐	A statement on whether measurements were taken from distinct samples or whether the same sample was measured repeatedly
☒	☐	The statistical test(s) used AND whether they are one- or two-sided<br>*Only common tests should be described solely by name; describe more complex techniques in the Methods section.*
☒	☐	A description of all covariates tested
☒	☐	A description of any assumptions or corrections, such as tests of normality and adjustment for multiple comparisons
☐	☒	A full description of the statistical parameters including central tendency (e.g. means) or other basic estimates (e.g. regression coefficient) AND variation (e.g. standard deviation) or associated estimates of uncertainty (e.g. confidence intervals)
☒	☐	For null hypothesis testing, the test statistic (e.g. *F*, *t*, *r*) with confidence intervals, effect sizes, degrees of freedom and *P* value noted<br>*Give P values as exact values whenever suitable.*
☒	☐	For Bayesian analysis, information on the choice of priors and Markov chain Monte Carlo settings
☒	☐	For hierarchical and complex designs, identification of the appropriate level for tests and full reporting of outcomes
☒	☐	Estimates of effect sizes (e.g. Cohen's *d*, Pearson's *r*), indicating how they were calculated

Our web collection on statistics for biologists contains articles on many of the points above.

Software and code

Policy information about availability of computer code

Data collection	Thermo Fisher Scientific Exactive Series Tune v2.10, Thermo Fisher Scientific Xcalibur Version 4.2.28.14, and Thermo Fisher Scientific EPU v3.6 and v3.10.
Data analysis	ChimeraX v1.7, CryoSPARC v4.4.1, v4.5, and v4.7.1, PHENIX 1.21.1-5286-000, Coot v0.9.8.

For manuscripts utilizing custom algorithms or software that are central to the research but not yet described in published literature, software must be made available to editors and reviewers. We strongly encourage code deposition in a community repository (e.g. GitHub). See the Nature Portfolio guidelines for submitting code & software for further information.

Data

Policy information about availability of data

All manuscripts must include a data availability statement. This statement should provide the following information, where applicable:

- Accession codes, unique identifiers, or web links for publicly available datasets
- A description of any restrictions on data availability
- For clinical datasets or third party data, please ensure that the statement adheres to our policy

The cryo-EM maps have been deposited in the Electron Microscopy Data Bank (EMDB) under accession codes EMD-52627 (soft-landed GroEL without ice), EMD-52626 (soft-landed GroEL with ice), EMD-52284 (soft-landed and rehydrated GroEL), EMD-52282 (plunge-frozen GroEL), EMD-52274 (soft-landed β-galactosidase without ice), EMD-52244 (soft-landed and rehydrated β-galactosidase (average)), EMD-52260 (soft-landed and rehydrated β-galactosidase (most similar)), EMD-52230 (plunge-frozen β-galactosidase) and EMD-55805 (soft-landed and rehydrated β-galactosidase (most

285 similar particles, as identified in a variability analysis without using the native particles as a reference)). The corresponding data set are accessible on EMPIAR (EMPIAR-12558, EMPIAR-12576, EMPIAR-12481, EMPIAR-12480, EMPIAR-12489, EMPIAR-12479, EMPIAR-12475). The atomic coordinates of the models have been deposited in the Protein Data Bank (PDB) under accession codes 9HKI (plunge-frozen β-galactosidase) and 9HLM (soft-landed and rehydrated β-galactosidase (most similar)).

Research involving human participants, their data, or biological material

Policy information about studies with human participants or human data. See also policy information about sex, gender (identity/presentation), and sexual orientation and race, ethnicity and racism.

Reporting on sex and gender	N/A
Reporting on race, ethnicity, or other socially relevant groupings	N/A
Population characteristics	N/A
Recruitment	N/A
Ethics oversight	N/A

Note that full information on the approval of the study protocol must also be provided in the manuscript.

Field-specific reporting

Please select the one below that is the best fit for your research. If you are not sure, read the appropriate sections before making your selection.

☒ Life sciences ☐ Behavioural & social sciences ☐ Ecological, evolutionary & environmental sciences

For a reference copy of the document with all sections, see nature.com/documents/nr-reporting-summary-flat.pdf

Life sciences study design

All studies must disclose on these points even when the disclosure is negative.

Sample size	The size of the cryo-EM data sets was chosen to allow for reconstructions with sufficient resolution to reveal enough structural detail necessary for our interpretation. Rehydration experiments were performed on two different samples for beta-galactosidase and on one sample for GroEL, with over 100 grid squares revitrified for each. This represents a sufficiently large number of samples, since the reproducibility of soft-landing and amorphous ice deposition had already been demonstrated previously. The laser flash melting and revitrification experiment, which is new in this study, was performed at least 100 times for each sample, ensuring reproducibility of this step.
Data exclusions	Single particle images were excluded following established 2D and 3D classification workflows.
Replication	The applicability of this method was tested on two different and independent protein systems: beta-galactosidase (2 samples) and GroEL (1 sample), with over 100 grid squares revitrified for each sample. All attempts at replication were successful.
Randomization	This is not relevant to our study as we perform reconstructions for the same protein sample, which was either plunge frozen in a conventional fashion or soft-landed and rehydrated.
Blinding	This is not relevant to our study as no group allocation was performed.

Reporting for specific materials, systems and methods

We require information from authors about some types of materials, experimental systems and methods used in many studies. Here, indicate whether each material, system or method listed is relevant to your study. If you are not sure if a list item applies to your research, read the appropriate section before selecting a response.

Materials & experimental systems

n/a	Involved in the study	
☒	☐	Antibodies
☒	☐	Eukaryotic cell lines
☒	☐	Palaeontology and archaeology
☒	☐	Animals and other organisms
☒	☐	Clinical data
☒	☐	Dual use research of concern
☒	☐	Plants

Methods

n/a	Involved in the study	
☒	☐	ChIP-seq
☒	☐	Flow cytometry
☒	☐	MRI-based neuroimaging

Plants

Seed stocks	N/A
Novel plant genotypes	N/A
Authentication	N/A
