# Supplementary material for: Cryo-EM sample preparation with soft-landing and laser flash melting
Source: Nat Commun. 2026 Sep 15;17:10850. doi: 10.1038/s41467-026-77723-6 (PMC13672294; doi:10.1038/s41467-026-77723-6)

nature portfolio

Peer Review File

Cryo-EM Sample Preparation with Soft-Landing and Laser Flash Melting

Corresponding Author: Dr Ulrich Lorenz

This file contains all reviewer reports in order by version, followed by all author rebuttals in order by version.

Attachments originally included by the reviewers as part of their assessment can be found at the end of this file.

Version 0:

Reviewer comments:

Reviewer #1

(Remarks to the Author)
In single particle cryo-EM, sample preparation has always been one of the major focuses of technological developments. Very different from the traditional approaches of freezing samples into thin vitreous ice, a recent development is the native soft-landing electrospray ion beam deposition, aiming to apply mass spectrometry method to gently deposit protein samples onto a supporting film at cryo-temperature. With many efforts in pursuing this new development, a major technical issue of this approach is the dehydration of the protein during the gas phase in mass spectrometry, which makes it unsuitable for preparing cryo-EM grids.

The manuscript by Barrass et al. described an approach to rehydrate the protein samples that were dehydrated during the soft-landing sample preparation by using laser introduced into column of an electron microscope to flash melting ice followed by refreezing. Laser induced flash melting is a new technique pioneered by Lorenz lab, senior author of this manuscript. Its potential applications have been explored, giving the higher cooling rate in refreezing sample into vitreous ice. In this manuscript, authors explored the possibility of re-hydrate dehydrated proteins in grids prepared by electrospray ion beam deposition by applying laser induced flash melting. The rationale of the approach is clear. At frozen condition, dehydrated proteins surrounded with vitreous ice are not rehydrated. But flash melting of ice provides a short time window for dehydrated proteins to be rehydrated by absorb waters from melted surroundings. Interesting findings presented in this manuscript include that, with b-galactosidase as an example, a small portion of proteins can be rehydrated almost entirely to its native conformation. Apparently, rehydration within a protein is not uniformly. The study reveals some interesting details in process of protein rehydration, which to me are quite intriguing and interesting. Overall, findings of this study are interesting in terms of providing experimental evidence that help to understand rehydration process of dehydrated proteins.

Overall, I think the manuscript presented very interesting findings, but the authors should think more about the goal of this study. In terms of preferred orientation, there are many other experimental ways to deal with it. There are also other soft-landing preparation method that do not dehydrate proteins. In comparison, this approach, even it works, it would require significant efforts and may not be practical for routine application.

My detailed comments are listed below:
1) A major purpose of the manuscript reads as to rescue the soft-landing mass spectrometry method for cryo-EM sample preparation. In this regard, my own opinion is that this is not significant. Even it works, this approach of combining soft-landing (dehydration) followed by laser flash-melting (rehydration) is unlikely to able to become a method that is practical for routine application. There are other electrospray methods that have been developed to land protein samples on grids without dehydration and yield high-resolution structure determination, such as described in PMID: 28664529. With my next comment, I doubt this approach would rescue the electrospray soft-landing method for cryo-EM sample preparation. Therefore, the significance of the findings in this manuscript is not in its potential to rescue the electrospray/soft-landing. Instead, I found that revealing how dehydrated proteins are rehydrated is interesting in understanding this phenomenon in a more detailed way, and it is meaningful for protein science in general. Following up on this by authors or others, one may be able to uncover some interesting details or mechanisms of rehydration.
2) Beta-galactosidase is a very stable complex, and only a portion of the proteins could be rehydrated to its native conformations. Indeed, some types of proteins can be rehydrated after dehydration, but this may not be a general character

of many other proteins. I would doubt that some flexible complexes do not fall apart once dehydrated, or dehydration would not denature / unfold or destroy some proteins or macromolecular complexes, and rehydration, regardless how, could not restore proteins to its fully functional native states. With such doubts, it is always a try-and-error tests (but rather expansive test in terms of efforts).
3) I wonder if rehydration may be improved or more particles can be rehydrated by extending rehydration time longer. Giving the time constraint of laser induced flash melting, it might hard to extend the time much beyond the current level, 30us melting pulse. Could author try repeated flash melting of the same grid multiple times to see if rehydration can be more completed, such as fully rehydrated particles are more homogeneous in conformation, and more particles are rehydrated? Repeated flash melting of the same region of the grid could potentially give more time for protein to be further rehydrated. If the time required to rehydrate most proteins to their native states, instead of prolonging the melting time during one laser flash, repeating the same procedure multiple times may work. If not, why?
4) It is interesting that variability analysis separate rehydrated particle and native particles along the same principal component. What happen if a series of reconstructions be calculated along the PC1, all the way from left to right of PC1 shown in Fig. 3f, and run a movie? Will that show somehow the structural view of rehydration?

Yifan Cheng

Reviewer #2

(Remarks to the Author)
Barrass et al. describe the results of using flash melting with a laser in an attempt to fix particles soft landed onto EM grids using a mass spectrometer. The use of mass spectrometry as a preparative tool for cryoEM sample preparation is an emerging technology that holds considerable promise to accelerate structural biology. That said, numerous articles have shown that these soft landed particles are damaged (presumably via dehydration). Thus, while use of such a laser to rehydrate soft landed particles has already been described, the present study extends that work as the authors have achieved considerably higher throughput, allowing higher resolution reconstructions and study of multiple particle types. The study is well conducted, thorough, and the results are certainly interesting, moving the field of cryoEM grid preparation by soft landing forward. Given the enormous potential of this nascent technology, I believe this work is suitable for publication in Nature Communications provided the authors can address the points below:

Major Points:

1. My main concern is the claim that the authors obtain reconstructions “indistinguishable” from conventionally prepared samples. I commend the authors for making their reconstructions available to the reviewers; however, looking at the maps it is clear that while the “most similar” beta galactosidase reconstruction, formed using only ~1.7% of the particles, does approach the native structure, it is certainly not indistinguishable. In fact, aligning and scaling the two structures and flicking between them demonstrates that some residual compaction clearly remains. To demonstrate this I have uploaded two files (side_by_side.jpg and animation.gif)

Examining these images it is clear that not only is the “most similar” still slightly compacted, it is clearly extremely heterogeneous apart from the very core. Therefore, in my opinion this cannot be described as “indistinguishable” from the conventional approach, and in fact should still be described as partially compacted. Language about being able to fix the molecules, and the “most similar” being essentially the same as the conventional is present in too many places to list, I believe these should all be toned down considerably.

2. Linked to this, I have major concerns with how the subset for the “most similar” was created. This was done by combining the soft landed data with the conventional data then running variability analysis and selecting those particles from the landed data that overlap with the conventional data along PC1. This is somewhat analogous to just taking all the particles that best “correlate” with some external reference (i.e., the conventional sample). Given defined but randomly positioned damage, performing this procedure will result in an average that looks very similar to the provided reference, even though no individual molecule matches. Interestingly when comparing the “best” map with some of the maps obtained from different parts of the path, those maps appear to be more native in certain regions than the “most similar” map which I believe provides some evidence the “most similar” map is artifactual. To be more convincing, the authors would need to pull out the “most similar” map without any comparison to the conventional map. If they cannot do this, this strong potential for overfitting at least needs to be pointed out and discussed.

3. The authors themselves note that in terms of the damaged particles, resolution does not necessarily have a clear meaning, and the obtained maps look considerably lower than their measured resolution. As the subject of the paper concerns rehydrating the molecules to hopefully make them closer to the native structure, the authors should calculate the map-to-model FSC vs a native structure for all of the reconstructions shown in the paper, which provides a far better metric for how “correct” these structures are.

Other Points:

1. The paper says 1-2 nm of ice was added after deposition of particles. Supplement says <10 nm. Is this <10 nm the 1-2 nm?

2. Why deposit only 1-2 nm of ice on top of the particles? Might the preferential orientation in the rehydrated B-galactosidase

experiments be occurring because the particles are so close to the surface since they are only buried in 1-2 nm of ice? Have the authors tried adding a thicker layer of ice on top of the particles? Perhaps putting less ice under the particles and more on top might help the preferential orientation problem.

3. How long was the grid exposed to water vapor to grow the 25 nm of ice on each side of the grid?

4. How many laser rehydrated spots were imaged? Is each grid square only targeted with a single laser pulse? Is it possible to have more than one laser rehydration spot in a single grid square?

5. In each laser spot, typically how many holes could be imaged for rehydrated particles? Was there any difference in particle quality at different locations within the laser spot?

6. Why deposit ice on both sides of the grid? Is this an important step or does it simply happen because water vapor can reach both sides of the grid?

7. All data for B-galactosidase was acquired on the same microscope. However, the comparative data for GroEL was acquired with three different microscopes. Do the authors feel that caused any bias amongst the data? The plunge frozen and rehydrated were acquired with the same microscope and conditions.

8. Have the authors considered doing cryoET to determine the position of the particles within the ice layer before and after laser rehydration? It would be enlightening to know how far the particles move during laser rehydration.

Version 1:

Reviewer comments:

Reviewer #1

(Remarks to the Author)
As I mentioned in my previous review comments, I think this study presented some interesting findings that are worth to be published. In this revision, authors address my comments in the revised manuscript and rebuttal with some minor revision, without toning down conclusions and statements. While I still support publication of this study in Nature Communication, I would still think that some statements are too strong.

Abstract: “Here, we demonstrate that proteins can be returned to their native state by …”. The current structures shown in figure 2 and 3 demonstrate at best to the level of “partially returned to their native state”.

Same for the paragraph of the introduction, “Here, we demonstrate …”, again, the results at best showing “partially” native state.

Yifan Cheng

Reviewer #2

(Remarks to the Author)
We thank the authors for incorporating many of our suggestions into the revision. We believe this is a much improved manuscript that will be of great interest to the nature communications readership. Of particular interest is the map-to-model FSCs values that are considerably worse than the nominal resolution measured from the dataset. We believe that the map-to-model resolution is a better indicator of the reconstruction quality in this case, and should be emphasized throughout the paper. Additionally the map to model resolution must be calculated and cleary stated for the GroEL reconstructions.

We would also encourage the authors to include map-to-model resolution for all reconstructions even those which the authors do not claim to be near native states as we feel this provides an excellent benchmark for comparisons for the future, although we appreciate that they are likely to be low.

Finally, this discrepancy between the resolutions merits some further highlighting and discussion in the text.

We feel that the inclusion of this map-to-model comparison makes it far easier to judge the quality of the results, and would be happy to recommend publication if these points are addressed.

Version 2:

Reviewer comments:

Reviewer #2

(Remarks to the Author)
The authors have satisfactorily addressed my comments, and I recommend the manuscript for publication.

Open Access This Peer Review File is licensed under a Creative Commons Attribution 4.0 International License, which permits use, sharing, adaptation, distribution and reproduction in any medium or format, as long as you give appropriate credit to the original author(s) and the source, provide a link to the Creative Commons license, and indicate if changes were made.
In cases where reviewers are anonymous, credit should be given to 'Anonymous Referee' and the source.
The images or other third party material in this Peer Review File are included in the article's Creative Commons license, unless indicated otherwise in a credit line to the material. If material is not included in the article's Creative Commons license and your intended use is not permitted by statutory regulation or exceeds the permitted use, you will need to obtain permission directly from the copyright holder.
To view a copy of this license, visit https://creativecommons.org/licenses/by/4.0/

We would like to thank the reviewers for their careful reviews. We are attaching our responses below.

Reviewer #1

In single particle cryo-EM, sample preparation has always been one of the major focuses of technological developments. Very different from the traditional approaches of freezing samples into thin vitreous ice, a recent development is the native soft-landing electrospray ion beam deposition, aiming to apply mass spectrometry method to gently deposit protein samples onto a supporting film at cryo-temperature. With many efforts in pursuing this new development, a major technical issue of this approach is the dehydration of the protein during the gas phase in mass spectrometry, which makes it unsuitable for preparing cryo-EM grids.

The manuscript by Barrass et al. described an approach to rehydrate the protein samples that were dehydrated during the soft-landing sample preparation by using laser introduced into column of an electron microscope to flash melting ice followed by refreezing. Laser induced flash melting is a new technique pioneered by Lorenz lab, senior author of this manuscript. Its potential applications have been explored, giving the higher cooling rate in refreezing sample into vitreous ice. In this manuscript, authors explored the possibility of re-hydrate dehydrated proteins in grids prepared by electrospray ion beam deposition by applying laser induced flash melting. The rationale of the approach is clear. At frozen condition, dehydrated proteins surrounded with vitreous ice are not rehydrated. But flash melting of ice provides a short time window for dehydrated proteins to be rehydrated by absorb waters from melted surroundings. Interesting findings presented in this manuscript include that, with b-galactosidase as an example, a small portion of proteins can be rehydrated almost entirely to its native conformation. Apparently, rehydration within a protein is not uniformly. The study reveals some interesting details in process of protein rehydration, which to me are quite intriguing and interesting. Overall, findings of this study are interesting in terms of providing experimental evidence that help to understand rehydration process of dehydrated proteins.

Overall, I think the manuscript presented very interesting findings, but the authors should think more about the goal of this study. In terms of preferred orientation, there are many other experimental ways to deal with it. There are also other soft-landing preparation method that do not dehydrate proteins. In comparison, this approach, even it works, it would require significant efforts and may not be practical for routine application.

My detailed comments are listed below:
1) A major purpose of the manuscript reads as to rescue the soft-landing mass spectrometry method for cryo-EM sample preparation. In this regard, my own opinion is that this is not significant. Even it works, this approach of combining soft-landing (dehydration) followed by laser flash-melting (rehydration) is unlikely to able to become a method that is practical for routine application. There are other electrospray methods that have been developed to land protein samples on grids without dehydration and yield high-resolution structure determination, such as described in PMID: 28664529. With my next comment, I doubt this approach would rescue the electrospray soft-landing method for cryo-EM sample preparation. Therefore, the significance of the findings in this manuscript is not in its potential to rescue the electrospray/soft-landing. Instead, I found that revealing how dehydrated proteins are rehydrated is interesting in understanding this phenomenon in a more detailed way, and it is meaningful for protein science in general. Following up on this by authors or others, one may be able to uncover some interesting details or mechanisms of rehydration.

We have added a reference to the publication in question (“Electrospray-assisted cryo-EM sample preparation to mitigate interfacial effects”, DOI 10.1038/s41592-024-02247-0). Indeed, the electrospray sample preparation method presented there does not suffer the complication that the proteins need to be rehydrated, since the sample is deposited in small droplets. However, for the same reason this approach also does not provide any mass selectivity. Using a mass spectrometer to deposit the proteins may also make it possible in the future to couple the approach to ion mobility spectrometry and thus even select only certain conformational states. We do however agree that the future will have to show whether the additional technical effort involved in our approach is practical.

We have also added a comment to the conclusion, stressing that our work may open up new avenues for studying protein hydration.

2) Beta-galactosidase is a very stable complex, and only a portion of the proteins could be rehydrated to its native conformations. Indeed, some types of proteins can be rehydrated after dehydration, but this may not be a general character of many other proteins. I would doubt that some flexible complexes do not fall apart once dehydrated, or dehydration would not denature / unfold or destroy some proteins or macromolecular complexes, and rehydration, regardless how, could not restore proteins to its fully functional native states. With such doubts, it is always a try-and-error tests (but rather expansive test in terms of efforts).

We agree with this comment. In the conclusion, we discuss that future experiments will also have to show whether this approach can be generalized to a wide range of proteins. We have expanded on this point in the conclusion and discuss that some lyophilized particles cannot be successfully rehydrated. Our hope is that the dehydration of the electrosprayed particles is so rapid that irreversible misfolding and aggregation of the particles can be avoided. However, this remain to be seen.

3) I wonder if rehydration may be improved or more particles can be rehydrated by extending rehydration time longer. Giving the time constraint of laser induced flash melting, it might hard to extend the time much beyond the current level, 30us melting pulse. Could author try repeated flash melting of the same grid multiple times to see if rehydration can be more completed, such as fully rehydrated particles are more homogeneous in conformation, and more particles are rehydrated? Repeated flash melting of the same region of the grid could potentially give more time for protein to be further rehydrated. If the time required to rehydrate most proteins to their native states, instead of prolonging the melting time during one laser flash, repeating the same procedure multiple times may work. If not, why?

As we note in the conclusion of the paper, we have recently shown that the timescale of our experiments can be extended to hundreds of microseconds by enclosing the sample between two thin, vapor deposited membranes. We hope that in the future this will allow us to return a majority of the particles to the native state.

4) It is interesting that variability analysis separate rehydrated particle and native particles along the same principal component. What happen if a series of reconstructions be calculated along the PC1, all the way from left to right of PC1 shown in Fig. 3f, and run a movie? Will that show somehow the structural view of rehydration?

We have added such a movie and made changes to the manuscript accordingly.

Reviewer #2

Barrass et al. describe the results of using flash melting with a laser in an attempt to fix particles soft landed onto EM grids using a mass spectrometer. The use of mass spectrometry as a preparative tool for cryoEM sample preparation is an emerging technology that holds considerable promise to accelerate structural biology. That said, numerous articles have shown that these soft landed particles are damaged (presumably via dehydration). Thus, while use of such a laser to rehydrate soft landed particles has already been described, the present study extends that work as the authors have achieved considerably higher throughput, allowing higher resolution reconstructions and study of multiple particle types. The study is well conducted, thorough, and the results are certainly interesting, moving the field of cryoEM grid preparation by soft landing forward. Given the enormous potential of this nascent technology, I believe this work is suitable for publication in Nature Communications provided the authors can address the points below:

Major Points:

1. My main concern is the claim that the authors obtain reconstructions “indistinguishable” from conventionally prepared samples. I commend the authors for making their reconstructions available to the reviewers; however, looking at the maps it is clear that while the “most similar” beta galactosidase reconstruction, formed using only ~1.7% of the particles, does approach the native structure, it is certainly not indistinguishable. In fact, aligning and scaling the two structures and flicking between them

demonstrates that some residual compaction clearly remains. To demonstrate this I have uploaded two files (side_by_side.jpg and animation.gif)

Examining these images it is clear that not only is the “most similar” still slightly compacted, it is clearly extremely heterogeneous apart from the very core. Therefore, in my opinion this cannot be described as “indistinguishable” from the conventional approach, and in fact should still be described as partially compacted. Language about being able to fix the molecules, and the “most similar” being essentially the same as the conventional is present in too many places to list, I believe these should all be toned down considerably.

We have included an additional figure in the SI that makes this comparison easier. Figure S11 reveals that the most similar particles are not compacted.

We have changed the language to describe more precisely how the reconstruction of the most similar particles relates to the native structure.

2. Linked to this, I have major concerns with how the subset for the “most similar” was created. This was done by combining the soft landed data with the conventional data then running variability analysis and selecting those particles from the landed data that overlap with the conventional data along PC1. This is somewhat analogous to just taking all the particles that best “correlate” with some external reference (i.e., the conventional sample). Given defined but randomly positioned damage, performing this procedure will result in an average that looks very similar to the provided reference, even though no individual molecule matches. Interestingly when comparing the “best” map with some of the maps obtained from different parts of the path, those maps appear to be more native in certain regions than the “most similar” map which I believe provides some evidence the “most similar” map is artifactual. To be more convincing, the authors would need to pull out the “most similar” map without any comparison to the conventional map. If they cannot do this, this strong potential for overfitting at least needs to be pointed out and discussed.

We can indeed obtain a map that closely resembles the reconstruction of the “most similar” particles in Fig. 3D from a variability analysis that does not include native particles as a reference. One of the principal components obtained from such a variability analysis corresponds to an expansion of the particles. If we select the most expanded particles, we obtain a reconstruction that is similar to the map in Fig. 3D, albeit with a slightly worse FSC with the model of the native structure (9.0 compared to 7.3 for the map in Fig. 3D). We have added a more detailed discussion of this analysis to Supplementary Information G.

This also makes it unlikely that the map in Fig. 3D, which was obtained from the “most similar” particles in the variability analysis of Fig. 3F, is artifactual. As the reviewer points out, it is conceivable that some of the particles selected from the variability analysis do not actually match the native structure. For this to be the case, these particles would have to differ significantly from the native particles in one of the other principal components or would have to be misassigned by the algorithm. However, this does not appear to be the case for the majority of the particles. If they indeed had randomly distributed deformations, with none of them actually resembling the native structure, a poor B factor should result, which is not what we find.

3. The authors themselves note that in terms of the damaged particles, resolution does not necessarily have a clear meaning, and the obtained maps look considerably lower than their measured resolution. As the subject of the paper concerns rehydrating the molecules to hopefully make them closer to the native structure, the authors should calculate the map-to-model FSC vs a native structure for all of the reconstructions shown in the paper, which provides a far better metric for how “correct” these structures are.

We have added map-to-model FSCs for β-galactosidase. Note that for the low-resolution structures in Fig. 3A and 3B, the map-to-model FSCs are not meaningful. We therefore did not include them.

Other Points:

1. The paper says 1-2 nm of ice was added after deposition of particles. Supplement says <10 nm. Is this <10 nm the 1-2 nm?

We have fixed this inconsistency.

2. Why deposit only 1-2 nm of ice on top of the particles? Might the preferential orientation in the rehydrated B-galactosidase experiments be occurring because the particles are so close to the surface since they are only buried in 1-2 nm of ice? Have the authors tried adding a thicker layer of ice on top of the particles? Perhaps putting less ice under the particles and more on top might help the preferential orientation problem.

For the laser flash melting experiments, 50 nm of amorphous ice were deposited. We have clarified this point in the main text.

3. How long was the grid exposed to water vapor to grow the 25 nm of ice on each side of the grid?

As noted in Supplementary Information C, we have determined our deposition rate to be about 15 nm/min. To deposit a 50 nm thick layer, we therefore deposit for a little more than 3 min.

4. How many laser rehydrated spots were imaged? Is each grid square only targeted with a single laser pulse? Is it possible to have more than one laser rehydration spot in a single grid square?

We center the laser beam onto a grid square and revitrify an area of typically 4 × 4 or 5 × 5 holes with a single 30 μs laser pulse (Supplementary Information C). In total, we imaged about 200 revitrified areas for both GroEL and β-galactosidase.

It should be possible to revitrify several spots within one grid square. However, in the grid geometry that we are currently using, this would not seem to offer any advantage.

5. In each laser spot, typically how many holes could be imaged for rehydrated particles? Was there any difference in particle quality at different locations within the laser spot?

We typically revitrify 4 × 4 or 5 × 5 holes per grid square. The particles in the center of the laser focus reach higher temperatures and remain in liquid for longer than the particles at the edge of the revitrified area. Therefore, one would expect that the particles in the center of the laser focus should be more similar to the native state. Unfortunately, the datasets were recorded without saving the stage positions, so that we cannot perform this analysis. However, this will be an interesting point to check in the future.

6. Why deposit ice on both sides of the grid? Is this an important step or does it simply happen because water vapor can reach both sides of the grid?

Correct, it would be advantageous to only deposit water on the side of the amorphous carbon film that carries the particles, which was not possible in our setup at the time. In future experiments, we indeed want to do so.

7. All data for B-galactosidase was acquired on the same microscope. However, the comparative data for GroEL was acquired with three different microscopes. Do the authors feel that caused any bias amongst the data? The plunge frozen and rehydrated were acquired with the same microscope and conditions.

The data for the maps of GroEL after soft-landing and after embedding in amorophous (Fig. 2A,B) were indeed recorded on a different microscope. Since these reconstructions only serve to illustrate that after soft-landing, the proteins are strongly compacted, but not to extract any high-resolution information, this should not be of concern.

8. Have the authors considered doing cryoET to determine the position of the particles within the ice layer before and after laser rehydration? It would be enlightening to know how far the particles move during laser rehydration.

Indeed, cryoET may be helpful to answer how the particles are distributed across the thickness of the sample. This is something that we will want to look into in the future.

We would like to thank the reviewers for carefully reviewing our revised manuscript.

Reviewer #1

As I mentioned in my previous review comments, I think this study presented some interesting findings that are worth to be published. In this revision, authors address my comments in the revised manuscript and rebuttal with some minor revision, without toning down conclusions and statements. While I still support publication of this study in Nature Communication, I would still think that some statements are too strong.

Abstract: “Here, we demonstrate that proteins can be returned to their native state by …”. The current structures shown in figure 2 and 3 demonstrate at best to the level of “partially returned to their native state”.

Same for the paragraph of the introduction, “Here, we demonstrate …”, again, the results at best showing “partially” native state.

We have removed these statements. Our experiments suggest that it will be possible to fully return soft-landed proteins to their native state if they are given more time to rehydrate. But indeed, only a small fraction of proteins approaches a configuration close to the native state in the experiments we show here.

Reviewer #2

We thank the authors for incorporating many of our suggestions into the revision. We believe this is a much improved manuscript that will be of great interest to the nature communications readership. Of particular interest is the map-to-model FSCs values that are considerably worse than the nominal resolution measured from the dataset. We believe that the map-to-model resolution is a better indicator of the reconstruction quality in this case, and should be emphasized throughout the paper. Additionally the map to model resolution must be calculated and cleary stated for the GroEL reconstructions.

We would also encourage the authors to include map-to-model resolution for all reconstructions even those which the authors do not claim to be near native states as we feel this provides an excellent benchmark for comparisons for the future, although we appreciate that they are likely to be low.

Since our GroEL sample seems to contain a mixture of different states, which we cannot separate in the rehydrated samples, we cannot easily provide map-to-model FSCs. We had previously speculated about the nature of these states but have removed these assignments since they appear too tentative.

We have instead opted to provide map-to-map FSCs with the map of the native structure throughout the manuscript. We believe that this allows for an accurate comparison between soft-landed and native structures. For reference, we also provide map-to-model FSCs for β-galactosidase in Table S1.

Finally, this discrepancy between the resolutions merits some further highlighting and discussion in the text.

We have expanded the discussion and highlighted that the poor FSC indicates remaining distortions of the protein structure. We have also added a local map-to-map FSC in Fig. S12. It shows that some structural differences appear to occur around water-filled voids, suggesting that they have not fully re-expanded.

We feel that the inclusion of this map-to-model comparison makes it far easier to judge the quality of the results, and would be happy to recommend publication if these points are addressed.

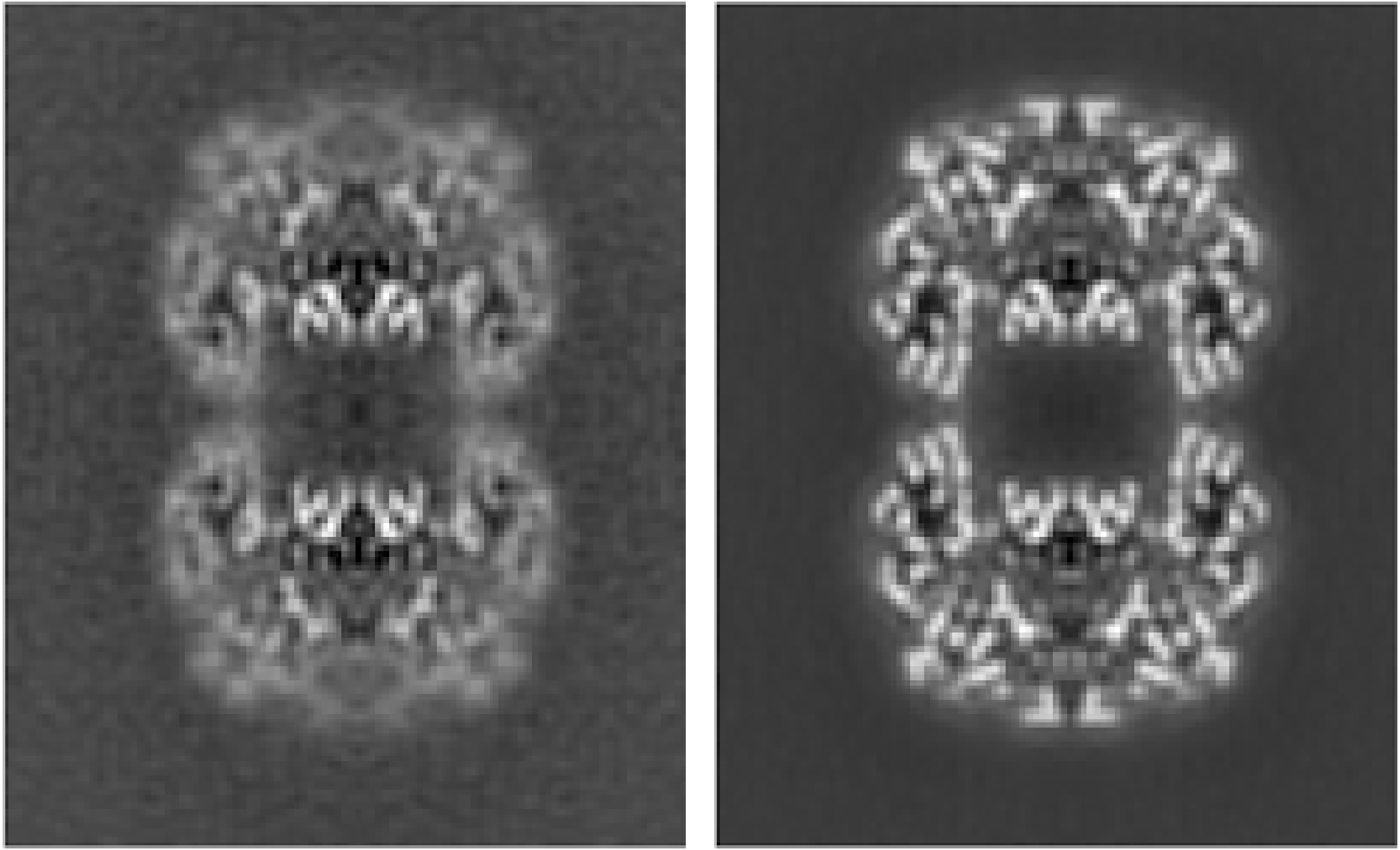

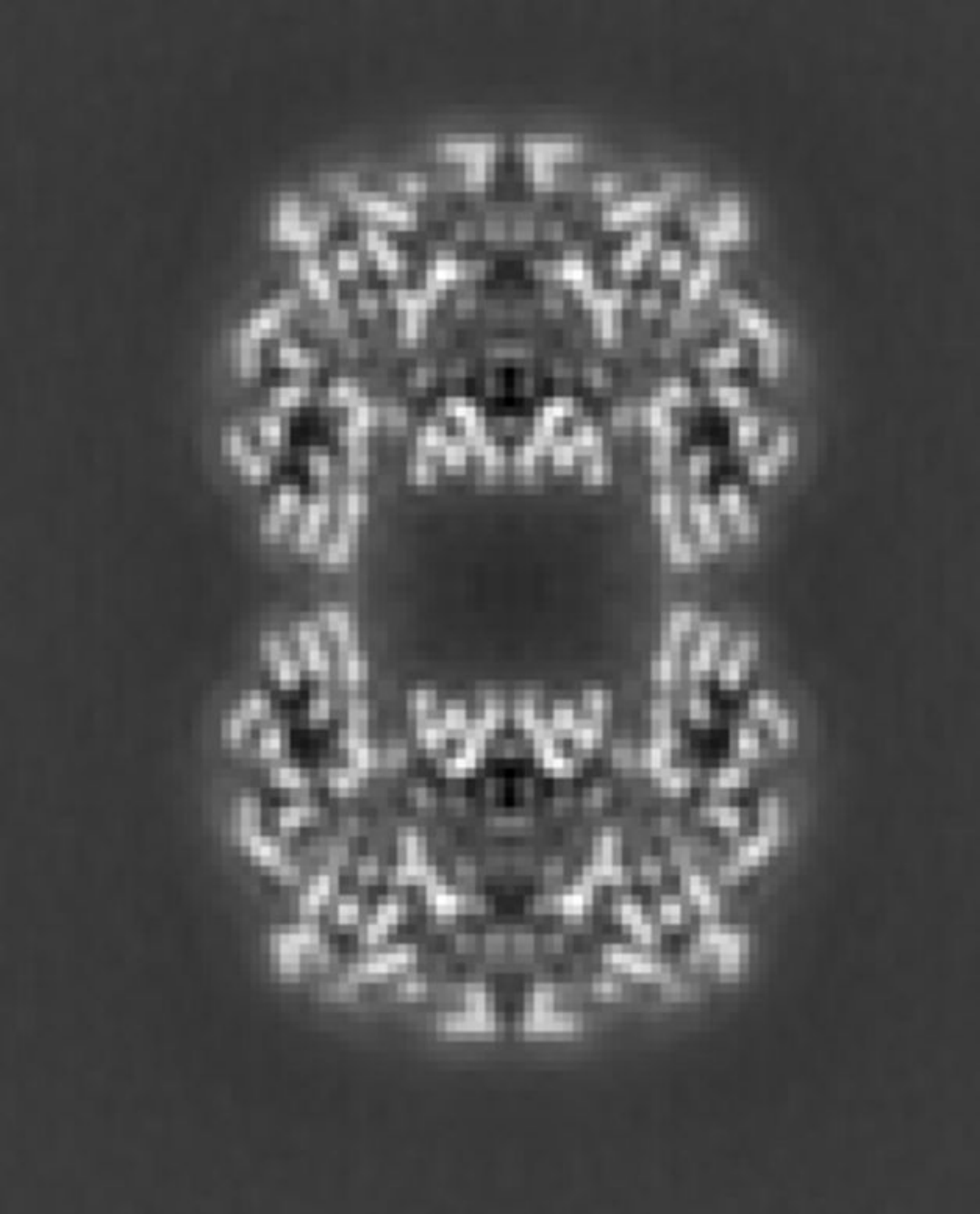

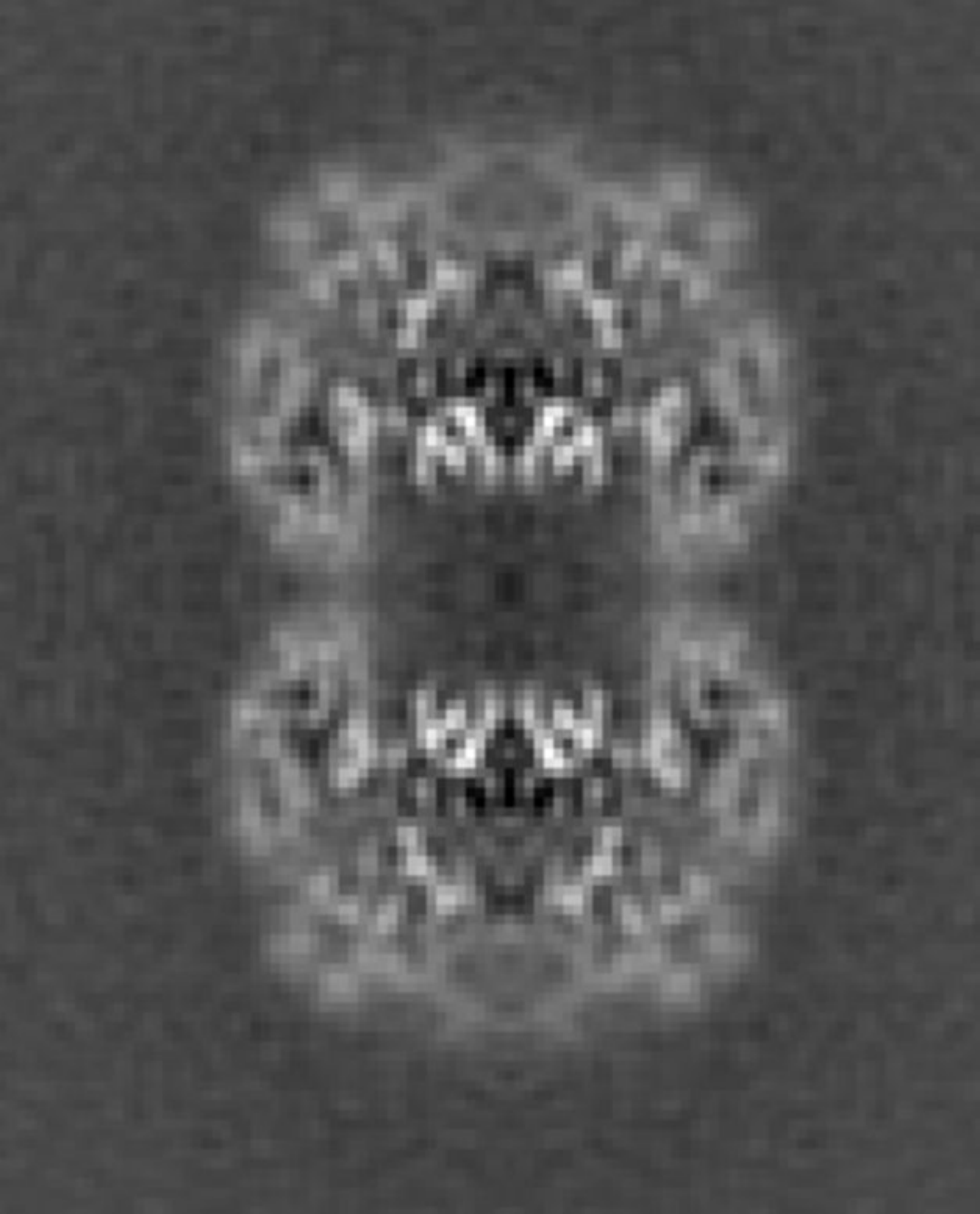

Supplement: Supplementary file 4 [file 41467_2026_77723_MOESM5_ESM.pdf]
